# Proteome Coverage after Simultaneous Proteo-Metabolome Liquid–Liquid Extraction

**DOI:** 10.1021/acs.jproteome.2c00758

**Published:** 2023-02-10

**Authors:** Alienke van Pijkeren, Anna-Sophia Egger, Madlen Hotze, Elisabeth Zimmermann, Tobias Kipura, Julia Grander, André Gollowitzer, Andreas Koeberle, Rainer Bischoff, Kathrin Thedieck, Marcel Kwiatkowski

**Affiliations:** † Institute of Biochemistry and Center for Molecular Biosciences Innsbruck, University of Innsbruck, Innsbruck, A-6020, Austria; ‡ Department of Analytical Biochemistry and Interfaculty Mass Spectrometry Center, Groningen Research Institute of Pharmacy, University of Groningen, Groningen, 9713 AV, The Netherlands; § Michael Popp Institute and Center for Molecular Biosciences Innsbruck (CMBI), University of Innsbruck, A-6020, Innsbruck, Austria; ∥ Laboratory of Pediatrics, Section Systems Medicine of Metabolism and Signaling, University of Groningen, University Medical Center Groningen, Groningen, 9713 AV, The Netherlands; ⊥ Department for Neuroscience, School of Medicine and Health Sciences, Carl von Ossietzky University Oldenburg, Oldenburg, 26129, Germany

**Keywords:** proteomics, metabolomics, sample preparation, simultaneous proteo-metabolomics, in-solution digest, SP3, mass spectrometry, label free quantification, bottom-up proteomics

## Abstract

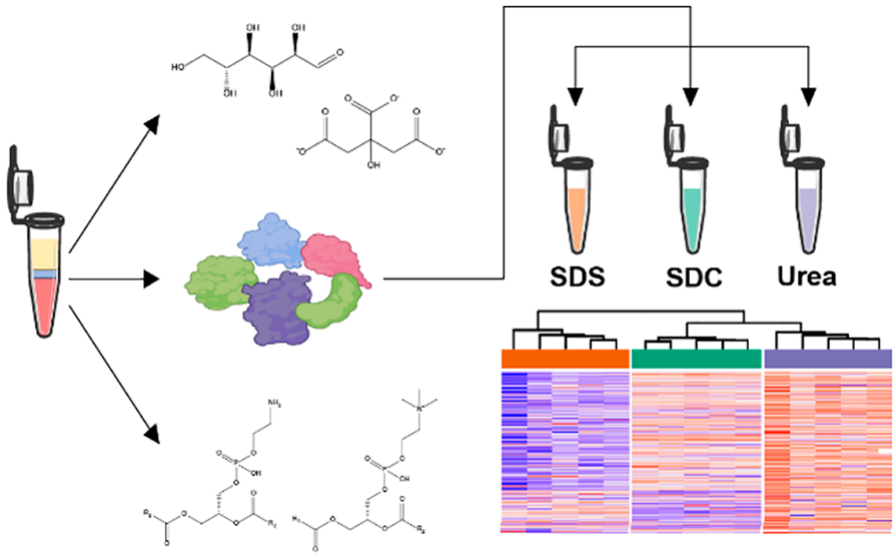

Proteomics and metabolomics are essential in systems biology, and simultaneous proteo-metabolome liquid–liquid extraction (SPM-LLE) allows isolation of the metabolome and proteome from the same sample. Since the proteome is present as a pellet in SPM-LLE, it must be solubilized for quantitative proteomics. Solubilization and proteome extraction are critical factors in the information obtained at the proteome level. In this study, we investigated the performance of two surfactants (sodium deoxycholate (SDC), sodium dodecyl sulfate (SDS)) and urea in terms of proteome coverage and extraction efficiency of an interphase proteome pellet generated by methanol–chloroform based SPM-LLE. We also investigated how the performance differs when the proteome is extracted from the interphase pellet or by direct cell lysis. We quantified 12 lipids covering triglycerides and various phospholipid classes, and 25 polar metabolites covering central energy metabolism in chloroform and methanol extracts. Our study reveals that the proteome coverages between the two surfactants and urea for the SPM-LLE interphase pellet were similar, but the extraction efficiencies differed significantly. While SDS led to enrichment of basic proteins, which were mainly ribosomal and ribonuclear proteins, urea was the most efficient extraction agent for simultaneous proteo-metabolome analysis. The results of our study also show that the performance of surfactants for quantitative proteomics is better when the proteome is extracted through direct cell lysis rather than an interphase pellet. In contrast, the performance of urea for quantitative proteomics was significantly better when the proteome was extracted from an interphase pellet than by direct cell lysis. We demonstrated that urea is superior to surfactants for proteome extraction from SPM-LLE interphase pellets, with a particularly good performance for the extraction of proteins associated with metabolic pathways. Data are available via ProteomeXchange with identifier PXD027338.

## Introduction

Multiomics technologies are essential in modern life sciences and systems biology. However, integrating analyses across different omics platforms is still a major analytical challenge, especially in terms of sample preparation. A common assumption is that the various omics sample preparation techniques are platform dependent and mutually exclusive. This is intriguing, as classical methods, which have been used in lipid analysis for decades, such as the Bligh and Dyer^1^ or the Folch^2^ extractions, provide simultaneous access to the lipidome, the metabolome, and the proteome. One reason for this discrepancy may be that sample preparation techniques used in metabolomics typically involve deproteinization steps to precipitate proteins by acid and/or organic solvents. Consequently, multiomics studies for which both the proteome and the metabolome need to be analyzed are often not done on the same sample, but rather two comparable samples are prepared independently, one for proteome and one for metabolome analysis.^3−6^ Methods for simultaneous proteo-metabolome extraction have been developed based on liquid–liquid extraction using methanol/chloroform^7−9^ or methanol/methyl-*tert*-butyl-ether.^10^ In all these approaches, an interphase pellet containing the proteins is generated, and mostly urea is used to solubilize the proteins for subsequent proteome analysis. However, there have been no studies on the influence of different extraction agents and buffers on protein extraction efficiency from interphase pellets, and consequently on proteome coverage in simultaneous proteo-metabolome liquid–liquid extraction (SPM-LLE) protocols. The choice of extraction agent is a critical step in multiomics analysis, as the chaotropic agents or surfactants used determine which part of the proteome is accessible for subsequent analysis.

There is also very limited information on whether and to what extent proteome coverages and extraction efficiencies differ between workup from an SPM-LLE interphase pellet and by direct cell lysis. Most studies focused on comparing the performance of chaotropic agents and surfactants in terms of proteome coverage and digestion efficiency of proteome extracts obtained by direct cell lysis, where the lysis buffer also acts as a proteome extraction buffer.^11−14^ Mass spectrometric based multiomics workflows are highly sophisticated multistep experiments combining different methods, instruments, and bioinformatics data processing workflows. The quality of multiomics experiments depends on the peculiarities and limitations of each step, with errors and/or biases of the individual steps propagating and accumulating throughout the experiment. Sample extraction is a critical step in the multiomics workflow, as chaotropic agents or surfactants determine which part of the proteome is available for subsequent analysis.

Here we describe a comparison of three different extraction agents commonly used in proteomics, the chaotropic agent urea, and the two surfactants sodium deoxycholate (SDC) and sodium dodecyl sulfate (SDS), with respect to proteome coverage, digestion, and proteome extraction efficiency of an interphase proteome pellet generated by methanol–chloroform based SPM-LLE. We also investigated how the performance of each extraction agent differs when the proteome is extracted from the SPM-LLE interphase pellet or through direct cell lysis, where the lysis buffer also acts as a proteome extraction buffer. For this study, we selected an immortalized human cell line (Human embryonic kidney 293T, HEK293T) as starting material and a label-free proteomics approach. Performance of the three proteome extraction agents for SPM-LLE, as well as between SPM-LLE and direct cell lysis, was compared based on qualitative and quantitative proteome coverage, digestion efficiency, physicochemical properties (e.g., size, charge characteristics, and hydrophobicity) of extracted proteins and their biological function. The results of this study will help researchers choose extraction agents for proteome extraction in a simultaneous proteo-metabolome analysis, as well as in conventional proteomics, according to the focus of the biological question and the relevant protein populations.

## Experimental Section

### Chemicals

HPLC-grade acetonitrile (ACN), methanol (MeOH), formic acid (FA), as well as Micro BCA Protein Assay Kit, Gibco Qualified FBS, and ammonium bicarbonate were obtained from Thermo Fisher Scientific (Dreieich, Germany). Dithiothreitol, iodoacetamide, HPLC-grade chloroform (CHCl_3_), urea, sodium deoxycholate (SDC), triethylammonium bicarbonate (TEAB), ethylenediaminetetraacetic acid (EDTA), Sera-Mag magnetic carboxylate modified hydrophilic and hydrophobic beads were obtained from Merck and Sigma-Aldrich (Munich, Germany). Dulbecco’s Modified Eagle Medium (DMEM), sodium dodecyl sulfate (SDS), sequencing grade modified trypsin, and HLB 1 cm^3^ (30 mg) extraction cartridges, were purchased from PAN Biotech (Aidenbach, Germany), Carl Roth (Karlsruhe, Germany), Promega (Walldorf, Germany), and Waters Oasis (Vienna, Austria), respectively. [U-^13^C]-labeled yeast extract was purchased from ISOtopic Solutions (Vienna, Austria). 1,2-Dimyristoyl-*sn*-glycero-3-phosphocholine (DMPC) and 1,2-dimyristoyl-*sn*-glycero-3-phosphoethanolamine (DMPE) were obtained from Avanti Polar Lipids (Alabaster, AL, USA), and 1,2,3-trimyristoyl-glycerol (TMG) was purchased from EDQM (Strasbourg, France).

[U-^13^C]-labeled yeast extract of *Pichia pastoris* (2 billion cells, ISOtopic solutions, Vienna, Austria) was reconstituted in 2 mL HPLC–H_2_O aliquoted and stored at −80 °C. 1,2- Dimyristoyl-*sn*-glycero-3-phosphocholine (DMPC, dissolved in CHCl_3_, Avanti Polar Lipids, Alabaster, AL, USA), 1,2-dimyristoyl-*sn*-glycero-3-phosphoethanolamine (DMPE, dissolved in CHCl_3_:MeOH 65:35, v/v; Avanti Polar Lipids, Alabaster, AL, USA), and 1,2,3-trimyristoyl-glycerol (TMG, dissolved in CHCl_3_, EDQM, Strasbourg, France) were combined and evaporated to dryness. The lipid film was taken up in a mixture of CHCl_3_:MeOH:H_2_O (65:35:8, v/v/v) leading to the final concentration of 0.2 mM for each standard. The lipid standard was aliquoted and stored under argon at −80 °C. All experiments were performed using five independent experiments (biological replicates).

### Cell Culture and SPM-LLE

One million HEK293T cells were seeded in 6 well plates and grown for 48 h at 37 °C and 5% CO_2_ atmosphere in high-glucose (*c* = 4.5 g/L) DMEM with FBS. The cells were washed three times with ice-cold PBS solution (pH 7.4). For extraction of extracellular metabolites, 50 μL of the medium was transferred into a reaction vial (Eppendorf low binding tube, 1.5 mL, Eppendorf, Hamburg, Germany) containing 450 μL ice-cold MeOH:H_2_O (8:1, v/v). The samples were vortexed for 20 s and centrifuged for 5 min at 16,100*g* and 4 °C. 200 μL of the supernatant were transferred into a reaction vial (Eppendorf low binding tube, 1.5 mL, Eppendorf, Hamburg, Germany) and dried using a rotary vacuum evaporator (Eppendorf Concentrator Plus, Eppendorf, Hamburg, Germany). The dried samples were stored at −80 °C for further LC-MS analysis. For simultaneous proteo-metabolome liquid–liquid extraction (SPM-LLE), 500 μL ice-cold methanol (MeOH) were added to the cells together with 20 μL of the [U-^13^C]-labeled yeast extract and 1 μL of the lipid standard (1,2-dimyristoyl-*sn*-glycero-3-phosphocholine (DMPC, *c* = 0.2 mM), 1,2-dimyristoyl-*sn*-glycero-3-phosphorylethanolamine (DMPE, *c* = 0.2 mM), 1,2,3-trimyristoyl-glycerol (TMG, *c* = 0.2 mM)), followed by 500 μL ice-cold water. Efficient cell lysis was ensured by shear forces generated by pipetting the methanol–water solution up and down 20 times using a P1000 pipet. Lysates were transferred into a reaction tube (Eppendorf low binding tube, 2 mL, Eppendorf, Hamburg, Germany), followed by addition of 500 μL ice-cold chloroform (CHCl_3_) and incubation for 20 min at 4 °C and 500 rpm on a thermo-shaker. Afterward, samples were centrifuged for 5 min at 4 °C and 16,000*g*. The polar and the nonpolar phase were transferred into two new and separate reaction vials (Eppendorf low binding tube, 1.5 mL, Eppendorf, Hamburg, Germany), evaporated to dryness using an Eppendorf Concentrator Plus (Eppendorf, Hamburg, Germany), and stored at −80 °C for further LC-MS analysis. The solid interphase pellet was evaporated to dryness using an Eppendorf Concentrator Plus (Eppendorf, Hamburg, Germany) and stored at −80 °C for proteome extraction.

### Protein Extraction from SPM-LLE Interphase Pellet Using Urea and Tryptic Digestion

The interphase pellets were dissolved in 60 μL urea buffer (8 M Urea, 100 mM ammonium bicarbonate (ABC), pH 8.3). The samples were diluted to a urea concentration of 2 M using 240 μL of 100 mM ABC (pH 8.3) and sonicated for 10 s at room temperature (Branson Ultrasonics Sonifier Model 250 CE, Thermo Fisher Scientific, parameters: constant duty cycle, output control: 2). Total protein amount was quantified by Pierce Micro BCA Protein Assay Kit (Thermo Fisher Scientific, Dreieich, Germany) following the vendor protocol using a 1:50 dilution of a sample aliquot (*V* = 10 μL) in HPLC-grade water (*V* = 490 μL). For tryptic digestion, a volume containing 100 μg of total protein was transferred to a new reaction tube and made up to a final volume of 100 μL with 100 mM ABC (pH 8.3). For reduction, 1.05 μL of a dithiothreitol containing reduction buffer (1 M DTT, dissolved in 100 mM triethylammonium bicarbonate (TEAB), pH 8.3) was added and samples were incubated for 30 min at 55 °C and 800 rpm on a thermo-shaker. For alkylation, 4.6 μL of iodoacetamide (IAA) containing alkylation buffer (0.5 M IAA, dissolved in 100 mM TEAB, pH 8.3) were added and samples were incubated for 30 min in the dark, followed by addition of 1.2 μL of reduction buffer to quench the alkylation reaction. Afterward, 102.2 μL of 100 mM ABC was added and proteins were digested for 16 h at 37 °C using 5 μg of trypsin (dissolved in trypsin resuspension buffer, Promega, Walldorf, Germany). Tryptic digestion was stopped by addition of 2.5 μL 100% formic acid. The samples were centrifuged for 5 min (16,000*g*, RT), and the supernatants were used for reversed phase solid phase extraction.

### Protein Extraction from SPM-LLE Interphase Pellet Using Sodium Deoxycholate (SDC) and Tryptic Digestion

The interphase pellets were dissolved in 300 μL SDC buffer (2% w/v SDC, 100 mM TEAB, pH 8.3). The samples were heated for 5 min at 98 °C, followed by sonication at room temperature (Branson Ultrasonics Sonifier Model 250 CE, Thermo Fisher Scientific, parameters: 1× 10 s, constant duty cycle, output control: 2). Total protein amount was quantified by Pierce Micro BCA Protein Assay Kit (Thermo Fisher Scientific, Dreieich, Germany) following the vendor protocol using a 1:50 dilution of a sample aliquot (*V* = 10 μL) in HPLC-grade water (*V* = 490 μL). For tryptic digestion, a volume containing 100 μg of total protein was transferred to a new reaction tube and made up to a final volume of 100 μL with 100 mM TEAB (pH 8.3). Reduction, alkylation, and quenching was performed as described above (see Protein Extraction from SPM-LLE Interphase Pellet Using Urea and Tryptic Digestion). Afterward, 102.2 μL of 100 mM TEAB was added and proteins were digested for 16 h at 37 °C using 5 μg of trypsin (dissolved in trypsin resuspension buffer, Promega, Walldorf, Germany). Tryptic digestion was stopped by addition of 2.5 μL 100% formic acid. The samples were centrifuged for 5 min (16,000*g*, RT) to remove the precipitated SDC. The supernatants were used for reversed phase solid phase extraction.

### Protein Extraction from SPM-LLE Interphase Pellet Using Sodium Dodecyl Sulfate (SDS) and Tryptic Digestion

The interphase pellets were dissolved in 300 μL sodium dodecyl sulfate (SDS) buffer (1% SDS, 100 mM ABC, pH 8.3). The samples were heated for 5 min at 98 °C, followed by sonication at room temperature (Branson Ultrasonics Sonifier Model 250 CE, Thermo Fisher Scientific, parameters: 1× 10 s, constant duty cycle, output control: 2), and total protein amount was quantified with a Pierce Micro BCA Protein Assay Kit (Thermo Fisher Scientific, Dreieich, Germany) following the vendor instructions using a 1:50 dilution of a sample aliquot (*V* = 10 μL) in HPLC-grade water (*V* = 490 μL). 100 μg of total protein was used for tryptic digestion using single-pot solid-phase-enhanced sample preparation (SP3) procedure.^15^ For this purpose, 50 μL each of carboxylate-modified hydrophilic and hydrophobic beads (Sera-Mag, Merck, Sigma-Aldrich, Munich, Germany) were combined to a final concentration of 10 μg/μL. Reaction vials containing the beads were placed on a magnetic rack for 2 min after which the supernatant was removed. 100 μL HPLC–H_2_O were added to the beads and incubated for 30 s at RT to wash the beads. Reaction vials were incubated on a magnetic rack for 2 min, supernatant was removed, and beads were suspended in 100 μL HPLC–H_2_O. For each sample, 100 μL of beads (total amount of beads: 1 mg) was added to 100 μg of proteins dissolved in 100 μL lysis buffer (1% SDS, 100 mM ABC, pH 8.3) to reach a beads/protein ratio of 10:1 (w/w). 467 μL of acetonitrile (ACN) was added to the samples to reach a final concentration of 70% ACN. The samples were incubated for 18 min at RT, followed by an incubation on a magnetic rack for 2 min. Afterward, the supernatants were transferred into new reaction vials, and the remaining reaction vials containing the beads were kept for further sample preparation (bead fraction 1). 100 μL of a new mixed bead solution (total amount of beads: 1 mg) was added to the reaction vials containing the supernatants. The samples were incubated for 18 min at RT, followed by an incubation on the magnetic rack for 2 min. Supernatants were removed and the reaction vials containing the beads were kept (bead fraction 2). Both bead containing reaction vials (bead fraction 1, bead fraction 2) were washed twice by adding 200 μL of 70% ethanol, incubation at RT for 30 s, and incubation on a magnetic rack for 2 min. The supernatants were removed. Additional two washing steps were performed by adding 300 μL 100% ACN incubation at RT for 30 s and incubation on a magnetic rack for 2 min. After removing the supernatant from the last washing step, beads were resuspended in 50 μL 50 mM ABC (dissolved in HPLC–H_2_O, pH 8.3). For reduction, 0.5 μL 1 M dithiothreitol (DTT, dissolved in 100 mM TEAB, pH 8.3) was added to the samples followed by an incubation at 56 °C for 30 min. For alkylation, 2 μL 0.5 M iodoacetamide (IAA, dissolved in 100 mM TEAB, pH 8.3) was added to the sample, which was incubated for 30 min in the dark, followed by addition of 0.6 μL of reduction buffer to quench the alkylation reaction. Five μg of trypsin (dissolved in trypsin resuspension buffer, Promega, Walldorf, Germany) were added and the samples were incubated for 16 h at 37 °C. Digestion was stopped by addition of 1435 μL of 100% ACN to reach a final ACN concentration of 95%. Samples were vortexed and incubated for 8 min at RT. The supernatants were removed and the samples were washed twice with 200 μL ACN following the washing procedure described above. Finally, peptides were eluted from the beads by addition of 100 μL elution buffer (2% DMSO,1% FA, dissolved in HPLC–H_2_O). The samples were incubated on a magnetic rack for 2 min and the supernatants containing the eluted peptides from both bead fractions were combined to one sample. The supernatants were used for reversed phase solid phase extraction.

### Protein Extraction by Direct Cell Lysis Using Urea and Tryptic Digestion

Six well dishes were washed three times with 300 μL PBS. Cells were lysed by addition of 60 μL of urea containing buffer (8 M urea, 100 mM ABC, pH 8.3). The samples were diluted to a final urea concentration of 2 M using 240 μL of 100 mM ABC (pH 8.3) and sonicated for 10 s at room temperature (Branson Ultrasonics Sonifier Model 250 CE, Thermo Fisher Scientific, parameters: constant duty cycle, output control: 2). Quantification of total protein amounts using Pierce Micro BCA Protein Assay Kit (Thermo Fisher Scientific, Dreieich, Germany) and tryptic digestion were performed as described above (see Protein Extraction from SPM-LLE Interphase Pellet Using Urea and Tryptic Digestion).

### Protein Extraction by Direct Cell Lysis Using Sodium Deoxycholate (SDC) and Tryptic Digestion

Six well dishes were washed three times with 300 μL PBS. Cells were lysed by addition of 300 μL SDC buffer (2% w/v SDC, 100 mM TEAB, pH 8.3). The samples were heated for 5 min at 98 °C, followed by sonication at room temperature (Branson Ultrasonics Sonifier Model 250 CE, Thermo Fisher Scientific, parameters: 1× 10 s, constant duty cycle, output control: 2). Quantification of total protein amounts using Pierce Micro BCA Protein Assay Kit (Thermo Fisher Scientific, Dreieich, Germany) and tryptic digestion were performed as described above (see Protein Extraction from SPM-LLE Interphase Pellet Using Urea and Tryptic Digestion).

### Protein Extraction by Direct Cell Lysis Using Sodium Dodecyl Sulfate (SDS) and Tryptic Digestion

Six well dishes were washed three times with 300 μL PBS. Cells were lysed by addition of 300 μL SDS buffer 1% SDS, 100 mM ABC, pH 8.3). The samples were heated for 5 min at 98 °C, followed by sonication at room temperature (Branson Ultrasonics Sonifier Model 250 CE, Thermo Fisher Scientific, parameters: 1× 10 s, constant duty cycle, output control: 2). Quantification of total protein amounts using Pierce Micro BCA Protein Assay Kit (Thermo Fisher Scientific, Dreieich, Germany), single-pot solid-phase-enhanced sample preparation (SP3), and tryptic digestion were performed as described above (see Protein Extraction from SPM-LLE Interphase Pellet Using Sodium Dodecyl Sulfate (SDS) and Tryptic Digestion).

### Reversed Phase Solid Phase Extraction (RP-SPE)

Samples were purified by RP-SPE prior to LC-MS analysis using OASIS HLB cartridges (Oasis HLB, 1 cc Vac Cartridge, 30 mg Sorbent, Waters, Manchester, UK) and a pressure manifold (Waters SPE Manifold, Waters, Manchester, UK). SPE cartridges were activated with each 1 mL of 100% methanol (MeOH), followed by 1 mL of 95% ACN, 1% FA, and equilibrated with 1 mL of 1% FA. The samples were adjusted to a final volume of 1 mL and a final concentration of 1% FA. Samples were loaded on SPE cartridges and washed twice with 1 mL 1% FA. Peptides were eluted with 1 mL of 70% ACN, 1% FA. The solvents of the eluates were evaporated and dried peptide samples were stored at −80 °C.

### Proteome Analysis by LC-MS/MS

Dried peptide samples were dissolved in 80 μL of 0.1% FA, and 1 μL of the samples were injected into a nanoultra pressure liquid chromatography system (Dionex UltiMate 3000 RSLCnano pro flow, Thermo Scientific, Bremen, Germany) coupled via electrospray ionization (ESI) to a tribrid Orbitrap mass spectrometer (Orbitrap Fusion Lumos, Thermo Scientific, San Jose, CA, USA). The samples were loaded (15 μL/min) on a trapping column (nanoE MZ Sym C18, 5 μm, 180 μm × 20 mm, Waters, Germany, buffer A: 0.1% FA in HPLC–H_2_O; buffer B: 80% ACN, 0.1% FA in HPLC–H_2_O) with 5% buffer B. After sample loading the trapping column was washed for 2 min with 5% buffer B (15 μL/min) and the peptides were eluted (250 nL/min) onto the separation column (nanoEase MZ PST CSH, 130 A, C18 1.7 μm, 75 μm × 250 mm, Waters, Germany; buffer A: 0.1% FA in HPLC–H_2_O; buffer B: 80% ACN, 0.1% FA in HPLC–H_2_O). The peptides were separated using a total gradient of 110 min. First, peptides were separated using a gradient from 5% B to 37.5% B in 90 min, followed by 37.5% B to 62.5% B in 25 min. The spray was generated from a steel emitter (Fisher Scientific, Germany) at a capillary voltage of 1850 V. MS/MS measurements were carried out in data dependent acquisition mode (DDA) using an HCD collision energy of 30% and top-speed scan mode. Every second a MS scan was performed over an *m*/*z* range from 350 to 1600, with a resolution of 120,000 fwhm at *m*/*z* 200 (maximum injection time = 50 ms, AGC target = 4 × 10^5^, internal calibration mode activated using ETD reagent for mass calibration). MS/MS spectra were recorded in the ion trap (rapid scan mode, maximum injection time = 50 ms, AGC target = 1 × 10^4^, quadrupole isolation width: 0.8 Da, intensity threshold: 1 × 10^4^). Precursors were excluded from DDA analysis for 60 s.

### Bioinformatics Data Processing of Proteome LC-MS/MS Data

LC-MS/MS raw data were processed and quantified with MaxQuant (version 1.6.5.0). Peptide and protein identification were carried out with Andromeda. LC-MS/MS data was searched against human database (SwissProt, 20,431 entries, downloaded 19.08.2019, https://www.uniprot.org/) and a contaminant database (239 entries). For database search, a mass tolerance of 6 ppm was used for precursor ions recorded at MS1 using the Orbitrap and a fragment mass tolerance of 0.5 Da was used for fragment spectra acquired in the ion trap. For peptide identification, two missed cleavages were allowed, a carbamidomethylation of cysteines was used as a static modification, and oxidation of methionine residues and acetylation of protein N-termini were allowed as variable modifications. Peptides and proteins were identified with an FDR of 1%. Proteins were quantified with the MaxLFQ algorithm considering only unique peptides, a minimum ratio count of two unique peptide and match between runs. The postprocessing of the data was performed in R (version 4.0.3) and RStudio (version 1.4.1106). Statistical analysis to compare protein yields and number of identified protein groups was done by two-sided *t*-tests using rstatix package (https://cran.r-project.org/web/packages/rstatix/index.html). Eulerr package (https://cran.r-project.org/web/packages/eulerr/index.html)^16^ was used to generate Venn diagrams. For differential proteome analyses, the MaxQuant output files “proteingroups.txt” and “modificationspecificpeptides.txt” were used, which contained the quantified LFQ-values for the identified protein groups. LFQ values were log2-transformed and normalized to the median for each sample (normalized protein group intensities). For principal component analysis (PCA), the normalized protein group intensities of the proteins reproducibly quantified in all samples were used as an input. For volcano plots, two-tailed *t*-tests were performed for all protein groups and adjusted *p*-values were calculated using the Benjamini–Hochberg procedure using the rstatix package (https://cran.r-project.org/web/packages/rstatix/index.html). The R package “Peptides” (https://cran.r-project.org/web/packages/Peptides/index.html)^17^ was used to calculate theoretical physicochemical properties of protein groups and GO enrichment analysis was performed using the gprofiler2 package (https://cran.r-project.org/web/packages/gprofiler2/index.html). The r ggplot2 package was used for data visualization (https://cran.r-project.org/web/packages/ggplot2/index.html).^18^

### Analysis of Polar Metabolites and Lipids by Ion Chromatography-Single Ion Monitoring-Mass Spectrometry (IC-SIM-MS) and Multiple Reaction Monitoring Mass Spectrometry (LC-MRM-MS)

#### Polar Metabolites

The dried samples of the intracellular and extracellular methanol extracts were dissolved in 100 μL HPLC–H_2_O and further diluted either 1:50 with HPLC–H_2_O for the analysis of low abundant metabolites or 1:2000 for the analysis of high abundant metabolites. Four μL of each sample were injected into a high-performance ion chromatography (HPIC) system (Dionex ICS-6000, Thermo Scientific, Germering, Germany). The separation was conducted on a Dionex IonPac AS11-HC column (2 mm × 250 mm, 4 μm particle size, Thermo Scientific) equipped with a Dionex IonPac AG11-HC guard column (Thermo Scientific) at 35 °C. A potassium hydroxide (KOH) gradient was produced by an eluent generator with a KOH cartridge (Dionex EGC 500 KOH, Thermo Scientific) that was supplied with HPLC–H_2_O. For the separation, a flow rate of 380 μL/min and the following gradient were used: 0 mM KOH to 3 mM KOH in 3 min, 3 mM KOH to 10 mM KOH in 2 min, 10 mM KOH to 30 mM KOH in 15 min, 30 mM KOH to 50 mM KOH in 7 min, 50 mM to 85 mM KOH in 2 min. A Dionex AERS 500 suppressor was used to exchange potassium ions against protons in order to produce H_2_O instead of KOH. A makeup flow (MeOH, 2 mM acetic acid) was provided at a flow rate of 60 μL/min. A T-piece connected the IC-eluate and makeup flow with a heated electrospray ion source (HESI) of an Orbitrap HF-X mass spectrometer (Thermo Scientific, Bremen, Germany). The following HESI source parameters were used: HESI temperature: 400 °C, sheath gas: 50, auxiliary gas: 10, auxiliary gas temperature: 380 °C, spray voltage: 2,500 V, S-Lens RF: 40, ion transfer capillary temperature: 380 °C. MS analyses were performed in negative ion mode. Full MS spectra were recorded with a *m*/*z* scan-range of 80–520 *m*/*z* with a resolution of 60,000 fwhm at *m*/*z* 200, maximum injection time of 50 ms and an AGC target of 1 × 10^5^. Metabolite quantification was carried out in targeted single ion monitoring (SIM) mode. Targeted SIM was acquired with a resolution of 60,000 fwhm at *m*/*z* 200, maximum injection time of 118 ms, AGC target of 1 × 10^5^, and an isolation window 4 *m*/*z* centered around the targeted *m*/*z*. The targeted SIM windows that were used are listed in Table 1.

**Table 1:** List of Polar Metabolites and [U-^13^C]-Labeled Standards, Retention Times, m/z, and Charge [*z*] Used for IC-SIM-MS

mass [*m*/*z*]	charge [*z*]	start *t* [min]	end *t* [min]	metabolite
179.05611	[M–H]^−1^	1.0	3.5	Glucose
87.00877	[M–H]^−1^	2.5	6.0	Pyruvate
259.02244	[M–H]^−1^	7.0	10.0	Glucose-1-Phosphate
117.01933	[M–H]^−1^	9.0	12.5	Succinate
133.01425	[M–H]^−1^	9.5	12.0	Malate
259.02244	[M–H]^−1^	10.0	16.0	Glucose-6-Phosphate, Fructose-6-Phosphate^a^
145.01425	[M–H]^−1^	12.8	15.0	α-Ketoglutarate
115.00368	[M–H]^−1^	14.1	16.0	Fumarate
289.03301	[M–H]^−1^	16.7	18.7	Sedoheptulose-7-Phosphate
346.05581	[M–H]^−1^	15.0	19.5	Adenosine monophosphate
275.01736	[M–H]^−1^	22.0	24.0	6-Phosphogluconate
173.00916	[M–H]^−1^	27.5	29.5	Aconitic acid
166.9751	[M–H]^−1^	27.5	29.5	Phosphoenolpyruvate
191.01973	[M–H]^−1^	25.4	29.0	Citrate, Isocitrate^a^
426.02214	[M–H]^−1^	30.5	32.0	Adenosine diphosphate (ADP)
337.98095	[M–H]^−1^	28.5	32.5	Fructose-1,6-Bisphosphate
505.98847	[M–H]^−1^	32.2	34.0	Adenosine triphosphate (ATP)
185.07624	[M–H]^−1^	1.0	3.5	[U-^13^C]-Glucose
90.01883	[M–H]^−1^	2.5	6.0	[U-^13^C]-Pyruvate
265.04257	[M–H]^−1^	7.0	10.0	[U-^13^C]-Glucose-1-Phosphate
137.02767	[M–H]^−1^	9.5	12	[U-^13^C]-Malate
265.04257	[M–H]^−1^	10	16	[U-^13^C]-Glucose-6-Phosphate
150.03102	[M–H]^−1^	12.8	15	[U-^13^C]-α-Ketoglutarate
119.0171	[M–H]^−1^	14.1	16	[U-^13^C]-Fumarate
356.08936	[M–H]^−1^	15	19.5	[U-^13^C]-AMP
281.03749	[M–H]^−1^	22	24	[U-^13^C]-6-Phosphogluconate
169.98516	[M–H]^−1^	27.5	29.5	[U-^13^C]-Phosphoenolpyruvate
197.03986	[M–H]^−1^	25.4	29	[U-^13^C]-Citrate
436.05569	[M–H]^−1^	30.5	32	[U-^13^C]-ADP
344.00108	[M–H]^−1^	28.5	32.5	[U-^13^C]-Fructose-1,6-Bisphosphate
467.02945	[M–H]^−1^	32.2	34	[U-^13^C]-ATP

a Both isobaric metabolites were measured in the same SIM window, as they exhibited baseline separation in IC.

Metabolite quantification was carried out with TraceFinder 5.0 General Quan (Thermo Scientific, San Jose, CA, USA). For peak identification and integration, the Genesis algorithm was used with the following parameters: Peak Detection Strategy: Highest Peak; Peak Threshold type: Area; Threshold: 1; Smoothing: 3; S/N threshold: 3; Tailing Factor: 3. If necessary, peaks were adjusted manually. Data were further processed using R (version 4.0.3) and RStudio (version 1.4.1106). R package rstatix (https://cran.r-project.org/web/packages/rstatix/index.html) was used to calculate the mean and standard deviation of the peak areas of the metabolites. The coefficient of variation (CV) was calculated by dividing the standard deviation by the mean and normalizing by a factor of 100. Violin plots of CVs were generated with R package ggplot2 (https://cran.r-project.org/web/packages/ggplot2/index.html)^18^ and heat maps were generated with R package gplots (https://cran.r-project.org/web/packages/gplots/index.html).

#### Small Chain Acyl-CoA

After dissolving the dried intracellular methanol extracts in 100 μL HPLC–H_2_O, 50 μL were used for SPE and targeted MS analysis of small chain acyl CoA molecules using a 2-(2-Pyridyl)ethyl silica gel-based SPE column (Supelco, Merck, Sigma-Aldrich, Germany) and hydrophilic interaction liquid chromatography (HILIC) coupled to single ion monitoring (SIM) MS analysis.^19^ For SPE extraction, samples were filled up to 1 mL with equilibration buffer (45% ACN, 20% H_2_O, 20% Acetic Acid, 15% Isopropanol (v/v), pH 3). SPE columns were equilibrated with 1 mL of equilibration buffer (45% ACN, 20% H_2_O, 20% Acetic Acid, 15% Isopropanol (v/v), pH 3). After equilibration, samples were loaded onto the SPE column and washed with 1 mL of the equilibration buffer. Analytes were eluted from the SPE columns with 2 mL of MeOH/250 mM ammonium formate (4 + 1 v/v, pH 7). The eluates were dried using a rotary vacuum evaporator (Eppendorf Concentrator Plus, Eppendorf, Hamburg, Germany). The dried samples were dissolved in 40 μL of 50% ACN. For HILIC-SIM-MS analysis, 1 μL of sample was injected on an UHPLC system (Vanquish Flex Quarternary UHPLC System, Thermo Scientific, Bremen, Germany) equipped with an amide HILIC column (Aquity UPLC BEH Amide, 130 Å, 1.7 μm, 2.1 × 150 mm, Waters, Germany). The UPLC was coupled via an electrospray-ionization (ESI) source to a quadrupole Orbitrap (QExactive HF-X, Thermo Scientific, Bremen, Germany). HILIC separation was performed using a gradient from 95 to 50% B in 8 min, and then from 50 to 10% B in 2 min (A: 10 mM NH_4_Ac in H_2_O, pH 10; B: 95% ACN, 5% 10 mM NH_4_Ac in H_2_O, pH 10). SIM-MS analysis was carried out in positive mode using a resolution of 60,000 fwhm at 200 *m*/*z*, a maximum injection time of 80 ms and an AGC target of 5 × 10^4^, and the following SIM isolation windows: acetyl-CoA: *m*/*z* 810.1330 ± 15, propionyl-CoA: 824.1487 ± 15, malonyl-CoA: 854.1229 ± 15, succinyl-CoA: 868.1385 ± 15.

Data analysis was performed in TraceFinder 5.0 (Version 5.0.889.0, Thermo Scientific, Bremen, Germany). Peaks were fitted using the Genesis algorithm with the following parameters: percent of highest peak: 1, minimum peak height (signal/noise): 3, signal-to-noise threshold: 2, tailing factor: 1. Peak integration was manually corrected if necessary. Data were further processed using R (version 4.0.3) and RStudio (version 1.4.1106) as described above in the “polar metabolite” section.

#### Lipids

After SPM-LLE, lipid films were dissolved in MeOH (100 μL), centrifuged (21,100*g*, 4 °C, 5 min), diluted 1:20 with MeOH and subjected to LC-MRM-MS after an additional centrifugation step (21,100*g*, 4 °C, 5 min). Chromatographic separation of phospholipids (phosphatidylcholines (PC), lysophosphatidylethanolamine (LPE), and phosphatidylinositols (PI)) was performed using an ExionLC AD UHPLC system (Sciex, Framingham, MA, USA) as previously described.^20^ Briefly, samples (injection volume: 3 μL) were separated at 45 °C on an ACQUITY UPLC BEH C8 column (130 Å, 1.7 μm, 2.1 × 100 mm; Waters, Milford, MA, USA) at a flow rate of 0.75 mL/min using buffer A (95% ACN, 2 mM ammonium acetate in H_2_O) and buffer B (10% ACN, 2 mM ammonium acetate in H_2_O). Lipids were separated with a gradient from 75% buffer A to 85% buffer in 5 min, followed by an increase to 100% buffer A within 2 min and a subsequent isocratic elution for another 2 min. Applying the instrumental setup described above, triglycerides (TAG) were separated according to Espada et al.^21^ at 45 °C and a flow rate of 0.75 mL/min using a gradient consisting of buffer A (95% ACN, 2 mM ammonium acetate in H_2_O) and buffer B (isopropanol). In short, the initial composition (90% buffer A) was reduced from 90% to 70% within 6 min, which was succeeded by isocratic elution for 4 min. Eluted lipids were ionized by electrospray ionization (PC, LPE, PI: negative ion mode; TAG: positive ion mode) using a Turbo V ion source (Sciex, Framingham, MA, USA). Phospholipids were detected by MRM using a QTRAP 6500^+^ Mass Spectrometer (Sciex, Framingham, MA, USA) following fragmentation of [M+OAc]^−^ (PC) or [M–H]^−^ ions (LPE, PI) to fatty acid anions, as described before.^20^ For the simultaneous detection of PC, PE, and PI, the curtain gas was set to 40 psi, the collision gas to medium, the ion spray voltage to −4500 V, the temperature to 500 °C, the sheath gas to 55 psi, and the auxiliary gas to 75 psi. The declustering potential was set to −44 V (PC) or −50 V (PE, PI), the entrance potential to −10 V, the collision energy to −38 eV (PE), −46 eV (PC), or −62 eV (PI), and the collision cell exit potential to −11 V (PC, PI) or −12 V (LPE). TAGs were detected by a QTRAP 6500^+^ mass spectrometer (Sciex, Framingham, MA, USA) in MRM mode by fragmentation of [M+NH_4_]^+^ adduct to [M–fatty acid acyl]^+^ ions, without discriminating between fatty acyl positional isomers.^20,21^ In variation to the settings described in the references above, the curtain gas was set to 40 psi, the collision gas to low, the ion spray voltage to 5500 V, the heated capillary temperature to 400 °C, the sheath gas pressure to 60 psi, the auxiliary gas pressure to 70 psi, the declustering potential to 120 V, the entrance potential to 10 V, the collision energy to 35 eV, and the collision cell exit potential to 26 V. Lipid species were identified based on mass spectrometric information and retention behavior, which depends on the chain length and the degree of unsaturation of the acyl chains.^22,23^ DMPC, DMPE, and TMG were used as internal standards for the quantitative analysis of PCs, PEs, and TGs.^24−26^ For quantification, the average of both transitions (PC, PE, PI) or the (most intensive) species-specific transition (TAG, lysophospholipids) was used and normalized to the internal standard (DMPC for PC, PI; DMPE for LPE; TMG for TAG) as well as the protein concentration.^27^ The coefficient of variation (CV) was calculated using absolute analyte intensities or blank subtracted peak areas (DMPC, DMPE, TMG). Violin plots of CVs were generated with R package ggplot2 (https://cran.r-project.org/web/packages/ggplot2/index.html)^18^ and heat maps were generated with R package splits (https://cran.r-project.org/web/packages/gplots/index.html). The system was operated by Analyst 1.7.1 (Sciex, Framingham, MA, USA) and the obtained chromatograms were processed by Analyst 1.6.3 (Sciex, Framingham, MA, USA).

## Results and Discussion

### Access to Lipids and Polar Metabolites of Central Energy Metabolism by Liquid–Liquid Extraction with Chloroform and Methanol

**Figure 1:**
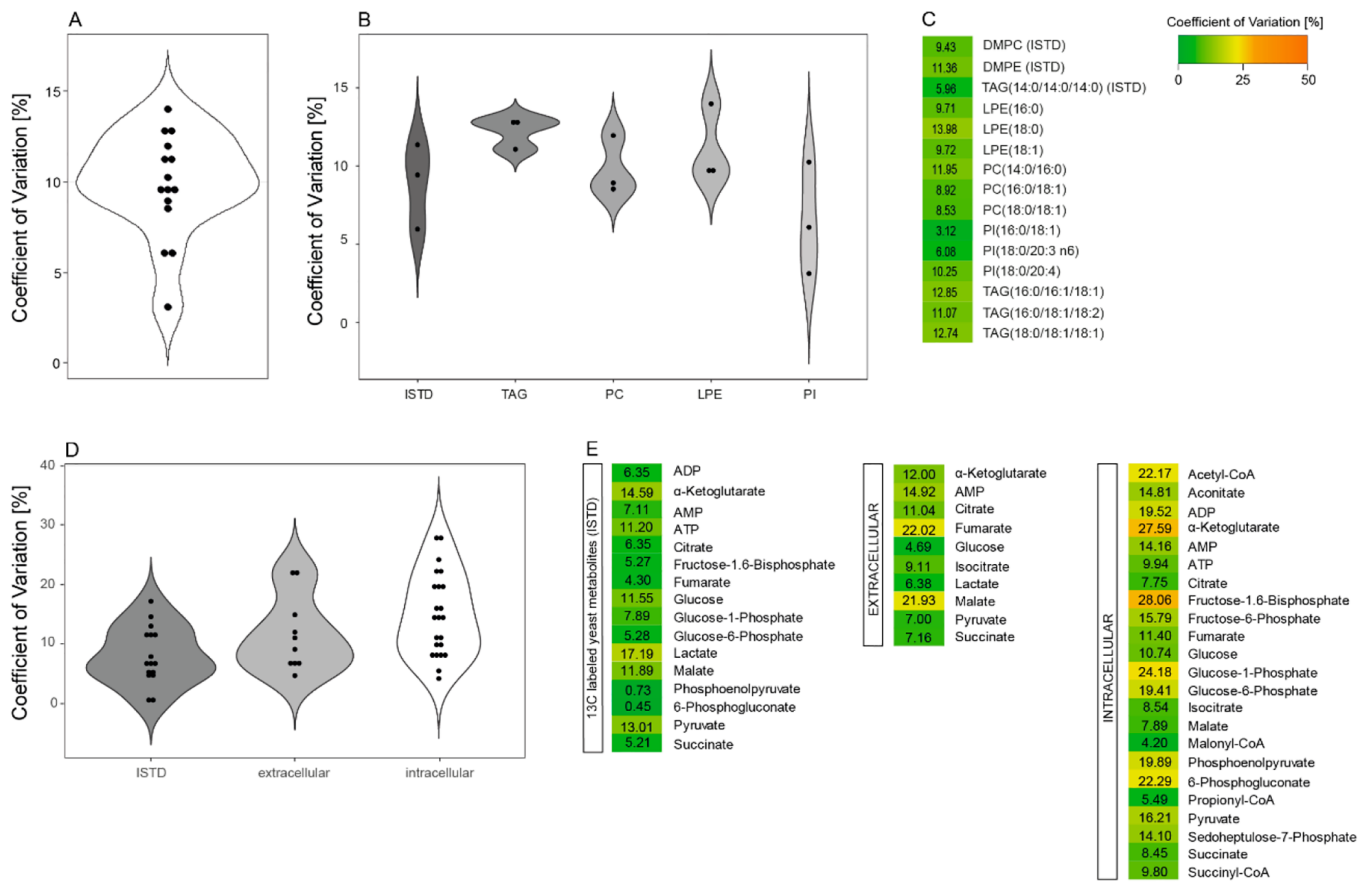
Quantification of selected lipids and polar metabolites extracted from the chloroform phase and methanol phase of a simultaneous proteo-metabolomic liquid–liquid extraction. (A, B) Violin plots showing the coefficient of variation [%] for all lipid analytes (A) and sorted based on lipid class and for the internal standards (ISTD, DMPC: 1,2-dimyristoyl-*sn*-glycero-3-phosphocholine, DMPE: 1,2-dimyristoyl-*sn*-glycero-3-phosphorylethanolamine, TMG: 1,2,3-trimyristoyl-glycerol) (B). (C) Color-coded representation of the coefficient of variation [%] of the different lipid analytes. LPE: lysophosphatidylethanolamine, PC: phosphatidylcholine, PI: phosphatidylinositol, TAG: triglyceride. (D) Violin plots showing the coefficient of variation [%] for internal standards (ISTD), extracellular and intracellular polar metabolites. (E) Color-coded representation of the coefficient of variation [%] of the ^13^C labeled yeast metabolites used as internal standards (ISTD), extracellular and intracellular polar metabolites. *n* = 5 independent experiments (biological replicates).

We used a MeOH–CHCl_3_-based SPM-LLE to extract the polar metabolome (MeOH phase), nonpolar metabolome including lipids (CHCl_3_ phase), and the proteome (interphase pellet). The only variation was in the processing of the proteomic interphase pellet, in which either urea, SDC or SDS were used as extraction agents to solubilize the interphase pellet. Although the main focus of this work was to evaluate the proteome accessibility of the interphase pellet, we also exemplarily quantified 12 lipids and 25 polar metabolites of central energy metabolism by targeted LC-MS to demonstrate the feasibility of simultaneous proteo-metabolome analysis. The 12 lipids covering triglycerides (TAG) and phospholipids (phosphatidylcholines (PC), lysophosphatidylethanolamine (LPE), and phosphatidylinositols (PI)) were quantified by targeted LC-MRM-MS. The 25 polar metabolites from central energy metabolism (glycolysis, the tricarboxylic acid (TCA) cycle, the pentose phosphate pathway, nucleotides, and small-chain acyl-coenzyme A (acyl-CoA) molecules) were quantified by targeted SIM-MS with either ion chromatography (IC) or hydrophilic interaction chromatography (HILIC). All lipids, including added internal standards (ISTD), were quantified by LC-MRM-MS with a coefficient of variation (CV) of less than 15% (Figure 1A–C). Results of the IC-SIM-MS analysis of polar metabolites showed that all internal standards (ISTD) were quantified with CV values below 15% (Figure 1D,E). For endogenous, intracellular polar metabolites, 5 out of 23 had CV values above 20% (α-ketoglutarate (CV = 27.6%), fructose-1,6-bisphosphate (CV = 28.1%), glucose-1-phosphate (CV = 24.2%), 6-phosphogluconate (CV = 22.3%), acetyl-CoA (CV = 22.17%)), whereas most metabolites (13 of 23) were quantified with CV values of less than 15%. Of 10 extracellular polar metabolites, 8 were quantified with CV values of less than 15%, while malate (CV = 21.9%) and fumarate (CV = 22.0%) had CV values slightly above 20%. These results confirmed that lipids and polar metabolites from central energy metabolism can be quantified using CHCl_3_–MeOH-based LLE, permitting simultaneous proteo-metabolome analysis.

### Total Protein Yield

To investigate proteome accessibility by MeOH–CHCl_3_-based SPM-LLE, we used buffers containing either urea (dissolved in 100 mM ammonium bicarbonate, pH 8.3), SDC (dissolved in 100 mM triethylammonium bicarbonate, pH 8.3), or SDS (dissolved in 100 mM ammonium bicarbonate, pH 8.3) to solubilize the interphase pellet. We first determined the total protein amount using a colorimetric bicinchoninic acid assay.

**Figure 2:**
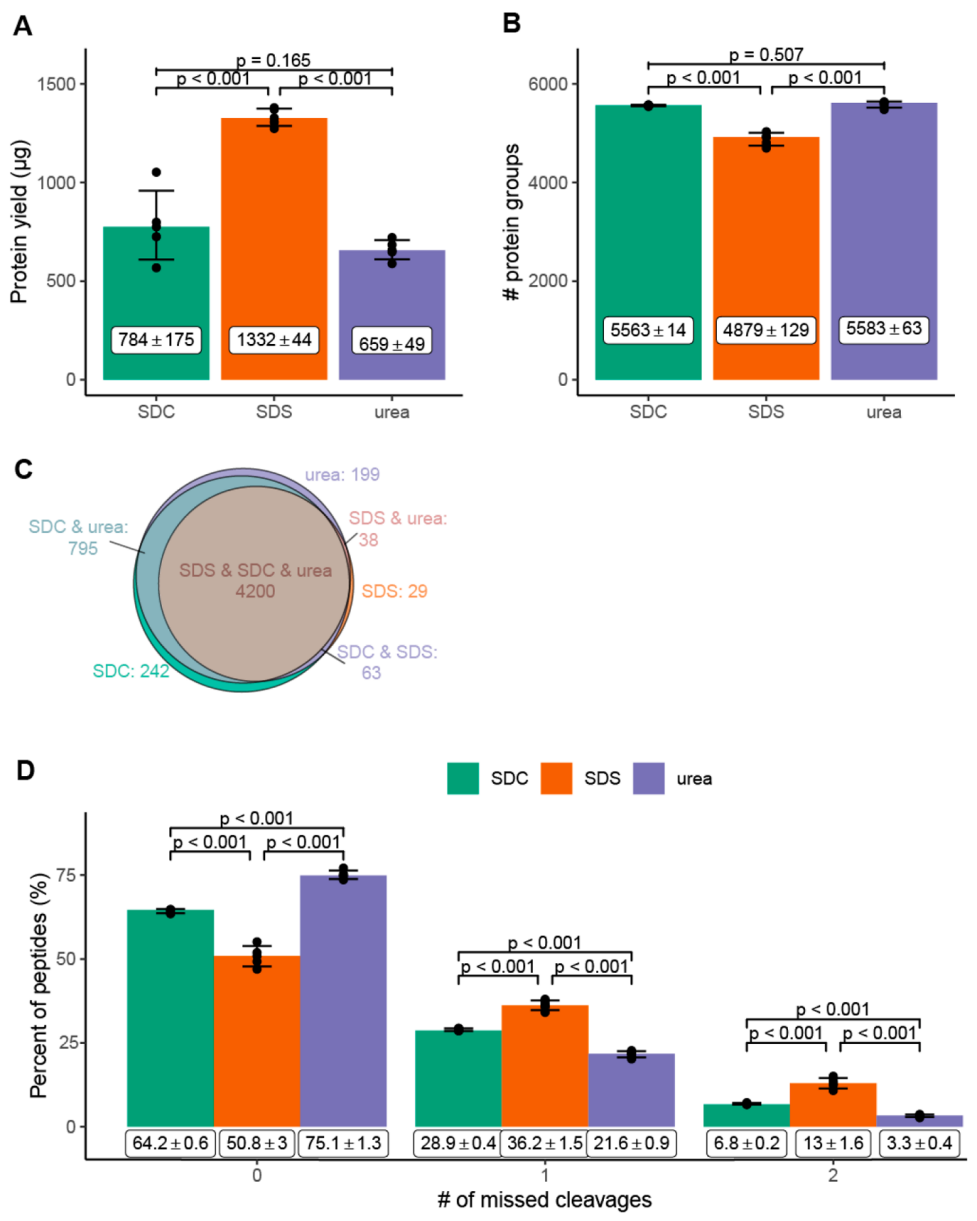
Protein yield (A), number of identified proteins (B, C), and relative percentage of missed cleavages after tryptic digestion (D) detected in the proteome solubilized from the interphase pellets. (A) Protein yield (μg) and (B) number of identified proteins from the interphase pellets solubilized by sodium deoxycholate (SDC, green), sodium dodecyl sulfate (SDS, orange), and urea (purple). (C) Number of proteins reproducibly identified in all replicates (*n* = 5) with the indicated buffer systems. (D) Relative percentage of missed cleavages after tryptic digestion of the interphase pellet extracted by SDC (green), SDS (orange), and urea (purple) based buffer systems. (A, B, D) Bar graph: mean with standard deviation, statistical analysis: two-tailed unpaired *t*-test. *n* = 5 independent experiments (biological replicates). Average number of cells: 4.3 × 10^6^ cells.

The average number of cells subjected to SPM-LLE was 4.3 × 10^6^ cells. The highest total protein yield was obtained with SDS (1332 μg ± 44 μg), followed by SDC (784 μg ± 175 μg) and urea (659 μg ± 49 μg) (Figure 2A). Solubilization of the proteome-containing interphase pellet with SDS and urea showed little variation compared with SDC. The variation for SDC was almost four times higher than for SDS and urea. Looking at the total amount of protein, our results show that SDS is the most efficient solubilizing agent to extract proteins from the SPM-LLE interphase pellet.

### Qualitative Analysis of the Solubilized Proteome

To compare the coverage of the proteome released from the SPM-LLE interphase pellet with urea, SDS, or SDC, we used a one-dimensional LC-MS/MS bottom-up label-free quantification (LFQ) proteomics approach. Equal amounts of proteins (*m* = 100 μg) extracted by urea, SDC, or SDS were digested with trypsin. For the samples solubilized by SDS, the single-pot solid-phase-enhanced sample preparation (SP3) procedure was used to remove SDS before tryptic digestion.^15^ SDC was removed after tryptic digestion by acid precipitation, and the urea concentration was reduced to less than 1 M before tryptic digestion by dilution. For all samples, the resulting peptides were desalted by reversed phase solid phase extraction (RP-SPE) and dried. The dried peptide–peptide samples were dissolved in equal volumes and subjected to LFQ LC-MS/MS analysis using an Orbitrap tribrid mass spectrometer (Fusion Lumos).

Each solubilizing agent yielded a reproducible number of identified proteins (Figure 2B). Urea (*n*_urea_ = 5583 ± 63) and SDC (*n*_SDC_ = 5563 ± 14) led to comparable numbers of protein identifications. In contrast, SDS (*n*_SDS_ = 4879 ± 129) yielded significantly lower protein identifications despite the better efficiency in solubilizing proteins from interphase pellets (Figure 2A). The lower number of identified proteins suggests the SP3 method is more prone to sample loss and/or introduces a bias toward certain proteins, so that coverage is overall less during sample preparation compared to acid precipitation (SDC) or dilution (urea). 75.5% of all proteins (*n* = 4200) were reproducibly identified with all three solubilizing agents (Figure 2C, Table S1). Varnavides et al. observed a similar overlap in protein identifications for urea, SDC, and SDS, when these agents were used in lysis buffers for direct proteome extraction from HeLa.^14^

The distributions of all identified proteins across the main gene ontology (GO) cellular component categories (membrane proteins, nuclear proteins, cytoplasmatic proteins, Figure S1A) were very similar for the three different extraction systems, and no differences were observed for the physicochemical properties of hydrophobicity, molecular weight, and isoelectric point (Figure S1B–D). For proteins exclusively identified in urea, SDC, or SDS, we did not observe differences in hydrophobicity (Figure S1E) and molecular weight (Figure S1F). Proteins identified exclusively in solubilization with SDS showed a shift to higher, more basic isoelectric points (Figure S1G). A potential explanation for this observation may be the anionic properties of the sulfate group of SDS. GO enrichment analysis did not reveal specific functional enrichment for proteins identified exclusively in SDS, SDC, and urea.

To investigate digestion efficiency for proteome extracts of SPM-LLE interphase pellets, we examined the effect of urea, SDC, and SDS on missed cleavages (MC) during tryptic digestion. Urea demonstrated the best digestion efficiency with the lowest number of missed cleavages (MC_0_ = 75.1%, MC_1_ = 21.6%, MC_2_ = 3.3%), followed by SDC (MC_0_ = 64.2%, MC_1_ = 28.9%, MC_2_ = 6.8%), and SDS (MC_0_ = 50.8%, MC_1_ = 36.2%, MC_2_ = 13%) (Figure 2D, Table S2–S4). Glatter et al.,^13^ León et al.,^12^ and Varnavides et al.^14^ each reported a higher tryptic digestion efficiency for SDC based direct cell lysis and proteome extraction than for urea. Our results suggest that urea has a higher digestion efficiency when the proteome is worked up from the pellet of the SPM-LLE interphase. A possible explanation could be that the solubilization and denaturation of the proteins of the SPM-LLE proteome pellet are more efficient than in direct cell lysis, making the proteins more accessible to tryptic digestion.

In summary, from a qualitative point of view, urea, SDS, and SDC-based proteome extractions from the SPM-LLE interphase pellet provide access to similar proteomes, with SDS showing a tendency to proteins with a higher isoelectric point. More obvious differences were observed for protein identification and tryptic digestion efficiency. SDS showed a significantly lower number of identified proteins compared to urea and SDC, and the highest number of missed cleavages. Using urea resulted in the highest digestion efficiency and lowest number of missed cleavages compared to surfactants.

### Quantitative Analysis of the Solubilized Proteome

In addition to the number of identified proteins and efficiency of tryptic digestion, an important question is whether the different agents provide different quantitative access to the proteomes extracted from the SPM-LLE interphase pellet. To answer this question, we performed a label free quantification (LFQ).

**Figure 3:**
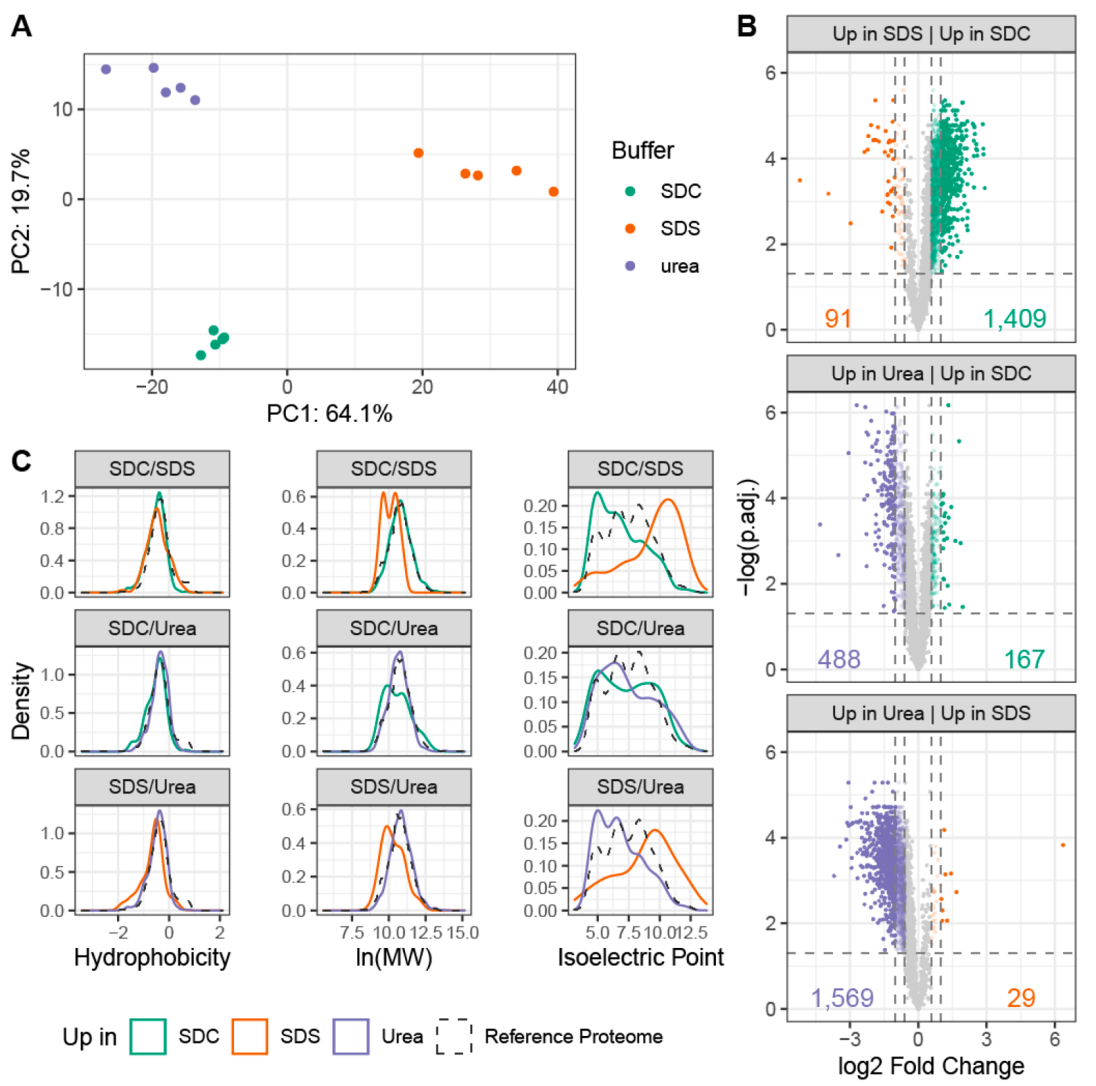
Quantitative analysis of proteins extracted from interphase pellets of simultaneous proteo-metabolomics liquid–liquid extractions (SPM-LLE). (A) Principal component analysis of proteins extracted from the SPM-LLE interphase pellets using sodium deoxycholate (SDC, green), sodium dodecyl sulfate (SDS, orange), or urea (purple). Protein abundance levels, reproducibly quantified in all independent experiments and conditions, were used as input for PCA. (B) Comparison of efficiency to extract proteomes from the SPM-LLE interphase pellet with SDC (green), SDS (orange), or urea (purple). Significance threshold for enrichment: adjusted *p*-value ≤0.05 (two-tailed unpaired *t*-test, Benjamini–Hochberg correction), fold change (FC) of 1.5: colored transparent dots, FC of ≥2: colored dots. (C) Physicochemical properties of proteins enriched in the SPM-LLE interphase pellets (colored solid lines, FC ≥ 1.5, adjusted *p*-value ≤0.05) as compared to the human reference proteome (SwissProt, uniprot.org, black dashed line). *n* = 5 independent experiments (biological replicates).

A principal component analysis (PCA) of the quantified protein intensities separated the independent experiments into distinct clusters for urea, SDC, and SDS (Figure 3A). These clusters were clearly separated in the first and second components, with the first component accounting for 64.1% of the summative variance and the second for 19.7%. Hierarchical clustering based on squared Euclidean distance measures using quantified protein intensities also separated the individual experiments into clusters assigned for the different extraction agents (Figure S2). Unsupervised analysis of the proteomics data indicates quantitative differences in extraction efficiency for urea, SDC, and SDS.

To get a more detailed overview of the extent to which the various reagents differ in their extraction efficiency, differentially extracted proteins were visualized in volcano-plots (Figure 3B). Proteins were considered significantly and differentially extracted at a threshold fold-change of at least 1.5 and an adjusted *p*-value below 0.05 (*t*-test, Benjamini–Hochberg correction). Based on these criteria, 1409 proteins were extracted more efficiently in SDC versus 91 in SDS. Comparing SDC with urea, 167 proteins were extracted more efficiently in SDC and 488 in urea. Comparing urea with SDS, 1596 proteins were extracted more efficiently in urea and 29 in SDS. By comparing the number of differentially extracted proteins between extraction reagents, urea provides the highest extraction efficiency in isolating proteomes from the SPM-LLE interphase pellet. The number of extracted proteins was 3-fold higher for urea compared to SDC, and 55-fold higher compared to SDS.

To further elucidate the extraction agent-specific differences systematically, we investigated the physicochemical properties of the differentially extracted proteome and performed GO enrichment analysis with these proteins. While differentially extracted proteomes did not exhibit differences in hydrophobicity, SDS extracted proteins of lower molecular weight more efficiently than SDC and urea (Figure 3C). Likewise, SDC showed a trend to extract proteins with a lower molecular weight more efficiently than urea. According to the qualitative analysis (Figure S1G), proteins extracted more efficiently in SDS showed a shift to higher, more basic isoelectric points. GO enrichment analysis of cellular components (GO:CC) showed significant enrichment of cytosolic as well as intracellular and membrane-bound organelle proteins in urea and SDC compared to SDS (Figure S3A,F). Compared to urea and SDC, ribosomal and ribonuclear proteins were enriched more efficiently in SDS (Figure S3B,E). Our quantitative analysis showed that SDS-based extraction resulted in specific enrichment of basic ribosomal and ribonuclear proteins (Figure S2, Figure S4). Sixty-nine of the 92 enriched proteins have an isoelectric point greater than 9, which explains the observed shift to higher isoelectric points for proteins extracted more efficiently with SDS (Figure 3C). While these ribosomal and ribonuclear proteins were underrepresented in the SDC proteome, this effect was less pronounced for the proteome extracted with urea (Figure S3B,D, Figure S4). One possible explanation for this observation is the anionic properties of the sulfate group of SDS, which disrupts electrostatic interactions between positively charged proteins and the negatively charged RNA, leading to a more efficient extraction of the ribosomal and ribonuclear proteins from the interphase pellet, which contains not only proteins but also nucleic acids. The more efficient extraction of these proteins with urea compared to SDC may be due to the fact that urea competes with the hydrogen bonds between the proteins and the RNA molecules by forming hydrogen bonds between the urea carbonyl and the amides in the protein backbone.^28,29^ Compared to SDC with its rather rigid sterane ring, urea is a relatively small molecule that can easily intercalate into protein-RNA complexes, which could explain the more efficient extraction of ribosomal and ribonuclear proteins from the interphase pellet.

In simultaneous proteo-metabolome analysis, the coverage of proteins related to metabolic pathways is of particular relevance for the integration of proteome and metabolome data. We therefore investigated the extraction efficiency for proteins involved in glycolysis and gluconeogenesis, the tricarboxylic acid (TCA) cycle, the pentose phosphate pathway (PPP), amino acid metabolism, and glycerolipid and glycerophospholipid metabolism. A qualitative comparison of the reproducibly identified proteins showed a similar coverage of all metabolic pathways with SDS, SDC, and urea (Figure S5). Quantitative analysis of extraction efficiency showed that proteins related to carbohydrate, lipid, and amino acid metabolism were best extracted from the interphase of SPM-LLE with urea (Table S5, Figure S4, Figure S6), whereas the lowest extraction efficiency was achieved with SDS. SDC showed higher efficiency than SDS in the extraction of proteins related to metabolic pathways.

### Proteome Coverage of the SPM-LLE Interphase Pellet Compared to Extraction by Direct Cell Lysis

The proteome is usually extracted from cells using a lysis buffer, which also serves as a proteome extraction buffer,^30,31^ and there is little information how proteome coverage and extraction efficiency differ between workup from an SPM-LLE interphase pellet and by direct cell lysis. We therefore directly extracted the proteome with urea-, SDC-, and SDS-containing lysis buffers, and performed an LFQ LC-MS/MS analysis to compare proteome extraction efficiency between SPM-LLE interphase pellet and direct cell lysis.

**Figure 4:**
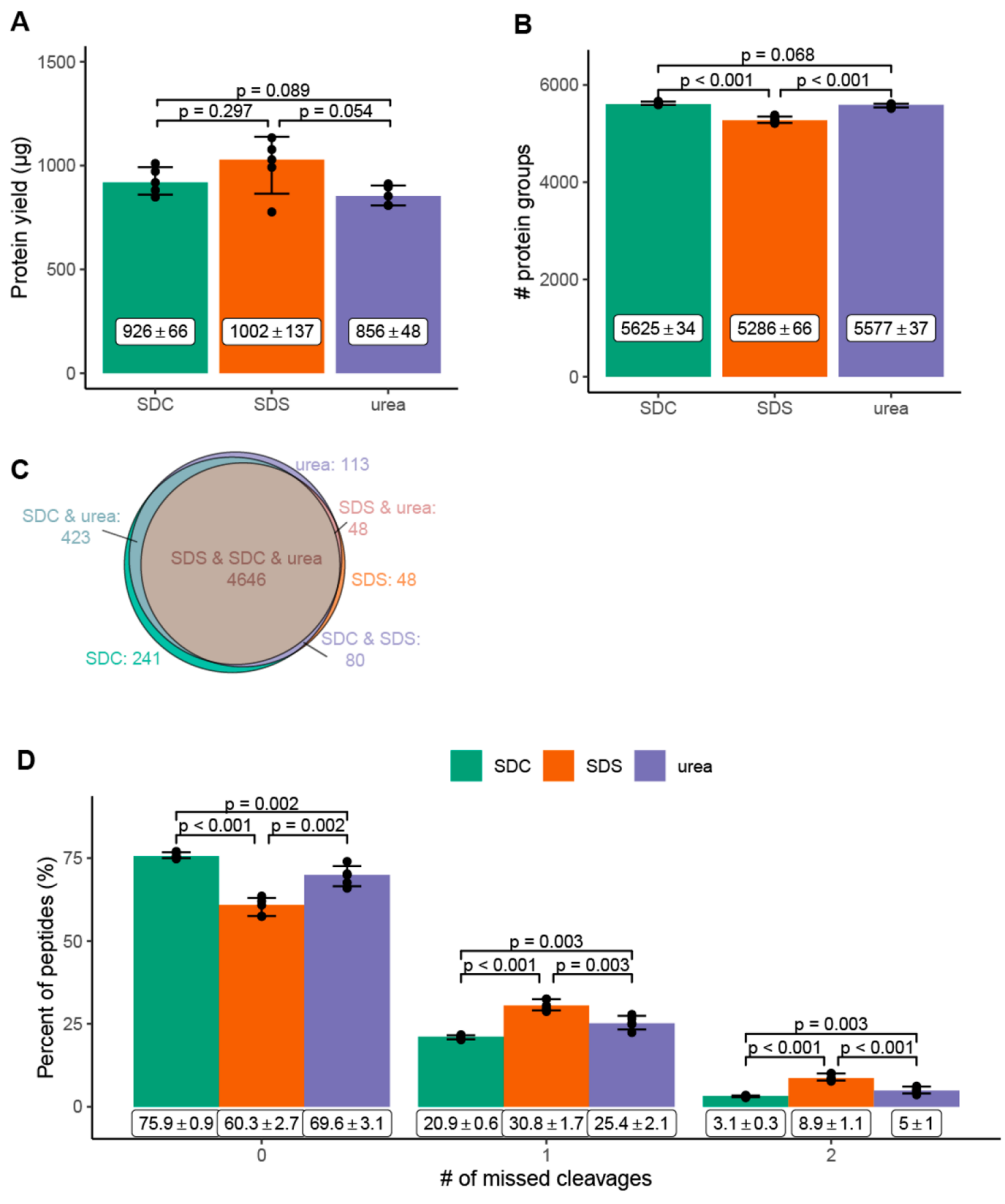
Protein yield (A), number of proteins identified (B, C), and relative percentage of missed cleavages after tryptic digestions (D) obtained by direct cell lysis. (A) Protein yield (μg) and (B) number of identified proteins by direct cell lysis using sodium deoxycholate (SDC), sodium dodecyl sulfate (SDS), and urea. (C) Number of proteins reproducibly identified in all biological replicates (*n* = 5) with the indicated extraction agents. (D) Relative percentage of missed cleavages after tryptic digestion of the proteome extracted by direct cell lysis using SDC (green), SDS (orange) and urea (purple) based buffer systems. (A, B, D) Bar graph: mean with standard deviation. Statistical analyses with two-tailed unpaired *t*-test. *n* = 5 independent experiments (biological replicates). Average number of cells: 4.3 × 10^6^ cells.

SDS provided the highest protein yield (1002 μg ± 137 μg) in direct proteome extraction (average number of cells: 4.3 × 10^6^ cells), followed by SDC (926 μg ± 66 μg) and urea (856 μg ± 48 μg) (Figure 4A). In contrast to solubilization of the interphase protein pellet, the use of SDS, SDC, and urea did not show significant differences in total protein yield during direct protein extraction from cells. The total protein yield was lower for SDS with direct cell lysis than with extraction from the interphase protein pellet, but for SDC and urea, direct cell lysis resulted in higher protein yields (Figure 2A, Figure 4A).

**Figure 5:**
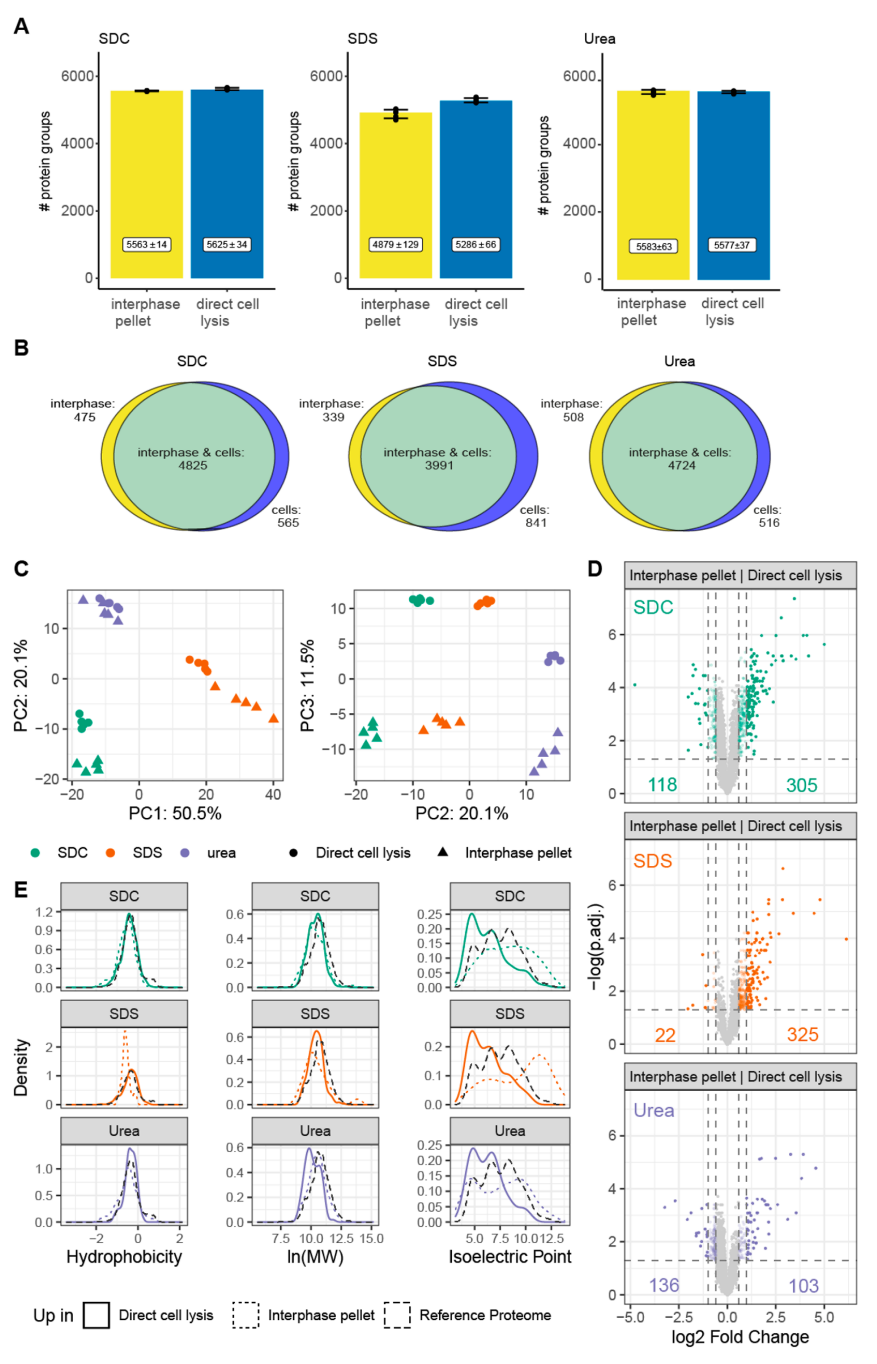
Comparison of proteins extracted from simultaneous proteo-metabolomics liquid–liquid extraction (SPM-LLE) interphase pellets versus direct cell lysis. (A) Number of identified proteins in the proteomes extracted from interphases of SPM-LLE (same data as shown in Figure 2B) and by direct cell lysis (same data as shown in Figure 4B) using SDC, SDS and urea. Mean with standard deviation. (B) Venn diagrams showing the number of proteins reproducibly identified in all replicates using SDC, SDS, and urea. Statistical analyses were performed using the two-tailed unpaired *t* test. *n* = 5 independent experiments (biological replicates). (C) Principal component analysis of proteins extracted from SPM-LLE interphase pellet (triangles) or direct cell lysis (circles). Protein abundance levels, reproducibly quantified in all independent experiments and conditions, were used as input for PCA. (D) Comparison of efficiency to extract proteomes from the SPM-LLE interphase pellets versus direct cell lysis. Significance threshold for enrichment: adjusted *p*-value ≤0.05 (two-tailed unpaired *t*-test, Benjamini–Hochberg correction), fold change (FC) of 1.5: colored transparent dots, FC of ≥2: colored dots. (E) Physicochemical properties of enriched proteins (FC ≥ 1.5, *p*-value ≤0.05). Proteins from SPM-LLE interphase pellets are represented with colored dashed lines, proteins from direct cell lysis are represented as colored solid lines. Physicochemical properties of the human reference proteome (SwissProt, uniprot.org) are shown with a black dashed line. Sodium deoxycholate (SDC, green), sodium dodecyl sulfate (SDS, orange), or urea (purple) containing buffer. *n* = 5 independent experiments (biological replicates).

For direct proteome extraction from cells, SDC yielded the highest number of identified proteins (*n*_SDC_ = 5625 ± 34), followed by urea (*n*_urea_ = 5577 ± 37) and SDS (*n*_SDS_ = 5286 ± 66) (Figure 4B). As for SPM-LLE, the use of SDS significantly reduced the number of identified proteins compared to SDC and urea, although the difference was not as pronounced for direct proteome extraction from cells as for the interphase pellet (Figure 2B). 82.8% of proteins (*n* = 4646) were reproducibly identified using urea, SDC, and SDS for direct cell lysis and proteome extraction (Figure 4C, Table S6). Comparison of the total number of proteins identified between direct cell lysis and SPM-LLE interphase pellets showed that the number of identified proteins was higher for SDC and SDS by direct cell lysis, whereas the number of identified proteins was higher for urea in SPM-LLE interphase pellets (Figure 5A). Most proteins were reproducibly identified in both direct cell lysis and from SPM-LLE pellets when SDC (92.5%), SDS (88.5%), or urea (91.9%) were used (Figure 5B). Taken together, we did not find significant differences in protein identification and proteome coverage for urea, SDC, and SDS between proteome extraction by direct cell lysis and SPM-LLE (Figure 5A,B). This confirms results by Nakayasu et al., who used urea for proteome extraction by direct cell lysis and interphase pellets after CHCl_3_–MeOH extraction.^9^ In our study, we obtained a similar overlap of protein identification for both direct cell lysis and SPM-LLE (Figure 2C, Table S1, Figure 4C, Table S6), Varnavides et al.^14^ reported for direct protein extraction using urea, SDC, and SDS. This suggests that there is no significant qualitative difference in proteome coverage when the proteome is isolated by direct cell lysis or an SPM-LLE interphase pellet.

Investigation of tryptic digestion efficiency for proteomes extracted by direct cell lysis showed that SDC provided the highest digestion efficiency with the lowest number of missed cleavages (MC_0_ = 75.9%, MC_1_ = 20.9%, MC_2_ = 3.1%), followed by urea (MC_0_ = 69.6%, MC_1_ = 25.4%, MC_2_ = 5.0%) and SDS (MC_0_ = 60.3%, MC_1_ = 30.8%, MC_2_ = 8.9%) (Figure 4D). The result is consistent with the previously reported higher digestion efficiency of SDC compared to urea.^12−14,32^ However, we found clear differences between SPM-LLE interphase pellets (Figure 2D) and direct extraction (Figure 4D) in the efficiency of digestion. The efficiency of the tryptic digest was improved by direct cell lysis for both SDC (Table S2) and SDS (Table S3), while urea showed better digestion efficiency when extracted from the SPM-LLE interphase pellet (Table S4). These results indicate that denaturation of proteins was more efficient during direct cell lysis than during SPM-LLE for SDC and SDS, making proteins more accessible to tryptic digestion in solution, whereas this was reversed for urea.

Next, we investigated whether extraction through direct cell lysis or SPM-LLE interphase pellets provides different quantitative access to proteomes for the different extraction agents. PCA analysis showed that the individual experiments clustered together, and that all conditions (extraction agents, SPM-LLE interphase pellet, proteome extraction by direct cell lysis) were clearly separated in the first (50.5%), second (20.1%), and third (11.5%) component (Figure 5C). Quantitative comparison of extraction efficiency between direct cell lysis and SPM-LLE showed that urea-based proteome isolation was more efficient from the SPM-LLE interphase pellet (*n* = 136) compared to direct cell lysis (*n* = 103) (fold change threshold 1.5, adjusted *p*-value <0.5, Benjamini–Hochberg correction) (Figure 5D). When we compared the number of proteins whose abundances differed significantly (adjusted *p*-value <0.05, Benjamini–Hochberg correction) regardless of the fold-change threshold, we found that considerably more proteins were extracted more efficiently from the SPM-LLE interphase pellet (*n* = 915) with urea than by direct cell lysis (*n* = 214). In contrast, both SDC and SDS extracted proteins more efficiently by direct cell lysis (SDC: 305, SDS: 325) than from SPM-LLE interphase pellets (SDC: 118, SDS: 22). We compared the extraction efficiency between SDC and urea, as previous studies reported better performance of SDC-based lysis buffers compared to urea for direct proteome extraction from cells.^12−14^ Consistently, SDC (*n* = 632) extracted more proteins efficiently than urea (*n* = 357) (Figure S7, Table S7). The results of our quantitative comparison of proteome extraction efficiency showed that the used surfactants achieved higher extraction efficiency through direct cell lysis, while higher extraction efficiency is achieved with urea when the proteome was isolated from SPM-LLE interphase pellets.

Finally, we compared the physicochemical properties and performed a GO analysis of the proteins that were more efficiently enriched by direct proteome extraction and SPM-LLE interphase pellets with urea, SDC, and SDS. We did not observe differences in hydrophobicity for the different extraction agents between direct cell lysis and SPM-LLE (Figure 5E). While differentially extracted proteomes showed no differences in molecular weight for SDS and SDC, urea showed a trend to extract proteins with lower molecular weight more efficiently in SPM-LLE than through direct cell lysis. We observed the most distinct differences in the distribution of isoelectric points. Extraction by direct cell lysis resulted in an enrichment of proteins with lower isoelectric points, while proteins with higher isoelectric points were extracted more efficiently from SPM-LLE interphases. This shift is explained by the higher coverage of ribosomal, ribonuclear, and mitochondrial proteins in SPM-LLE interphase pellets (Figure S8, Figure S9). In contrast, proteome extraction by direct cell lysis led to higher coverage of proteins assigned to extracellular exosomes and vesicles (Figure S8), which explained the shift to lower isoelectric points, as most of these proteins have an isoelectric point below seven. A possible explanation for this observation could be a loss of vesicles during the phase separation of SPM-LLE and a more efficient separation of nucleic acids from ribosomal and ribonuclear proteins during SPM-LLE. For proteins related to metabolic pathways, we did not observe clear differences between SPM-LLE and direct proteome extraction with SDC (Figure S10). While we observed higher extraction efficiency with SDS through direct proteome extraction from cells (Figure S11), urea extracted proteins related to carbohydrate, lipid, and amino acid metabolism significantly more efficiently from the SPM-LLE interphase pellet (Figure S12).

In summary, the results of our study show that proteome coverage between SDC, SDS, and urea-based extraction is similar and does not differ remarkably between direct cell lysis and workup from an SPM-LLE interphase pellet. However, proteome extraction efficiency differs significantly between direct cell lysis and SPM-LLE interphase pellets. For the surfactants SDS and SDC, higher extraction efficiency was achieved through direct cell lysis, while higher proteome extraction efficiency for urea was achieved by solubilizing SPM-LLE interphase pellets, including proteins related to metabolic pathways. All extraction agents solubilized proteins with low isoelectric points more efficiently by direct cell lysis. These were particularly proteins assigned to extracellular exosomes and vesicles. In contrast, proteins with higher isoelectric points were extracted more efficiently from the SPM-LLE interphase pellets. These were mainly ribosomal, ribonuclear, and mitochondrial proteins.

## Conclusion

To date, no studies have investigated the performance of chaotropic agents and surfactants for proteome extraction from SPM-LLE interphase pellets, and how proteome coverage and extraction efficiency differ between workup from an SPM-LLE interphase pellet and proteome extraction through direct cell lysis. To fill this gap, we examined the performance of three widely used proteome extraction agents (urea, SDC, SDS) to extract the proteome by SPM-LLE and direct cell lysis. In our study, SDS showed the lowest extraction efficiency for quantitative proteomics for both direct cell lysis and SPM-LLE interphase pellets. The lower extraction efficiency we observed could possibly be due to protein losses due to absorption by the carboxylate-modified hydrophilic and hydrophobic beads used for single-pot solid-phase-enhanced sample preparation (SP3). Varnavides et al. also reported that the bead-mediated protein pulldown could possibly lead to SP3-specific protein extraction.^14^ However, it should be noted that, despite the reduced performance, SDS-based proteome extraction in combination with SP3 leads to the enrichment of ribosomal and ribonuclear proteins, and could therefore be considered for the analysis of this specific subproteome. When comparing urea and SDC, the best performance was achieved in direct proteome extraction with a cell lysis buffer containing SDC. Similar results were previously reported by Glatter et al.,^13^ León et al.,^12^ and Varnavides et al.^14^ Beyond the works cited, our study showed that the performance of surfactants for quantitative proteomics is better when the proteome was extracted by direct cell lysis and not from an SPM-LLE interphase pellet. On the other hand, the performance of urea was significantly better for quantitative proteomics when the proteome was extracted from an interphase pellet than direct cell lysis. We demonstrated that urea is superior to surfactants for proteome extraction from SPM-LLE interphase pellets. The number of proteins extracted was 3-fold higher for urea than for SDC and 55-fold higher than for SDS, with a particularly good performance in extracting proteins related with metabolic pathways. Of the extraction agents tested here, urea is the most efficient for simultaneous proteo-metabolome analysis.

## Data Availability

The LC-MS data have been deposited to the ProteomeXchange Consortium via the PRIDE partner repository with the dataset identifier PXD027338.

## Supporting Information Available

Localization and physicochemical properties of proteins identified in SPM-LLE (Figure S1); relative abundances of all proteins quantified in SPM-LLE (Figure S2); GO analysis of proteins extracted more efficiently with SDC, SDS, and urea (Figure S3); relative abundances related to specific KEGG pathways (Figure S4); comparison of identified proteins that are related to metabolic pathways (Figure S5); extraction efficiencies for proteins related to metabolic pathways (Figure S6); quantitative comparison of proteins extracted by direct cell lysis using urea or SDC (Figure S7); GO analysis of proteins extracted more efficiently by direct cell lysis or SPM-LLE (Figure S8, Figure S9); differential analysis of extraction efficiencies for metabolic proteins (Figure S10, Figure S11, Figure S12); number of reproducibly identified proteins SPM-LLE and direct cell lysis (Table S1, Table S7); relative percentage of missed cleavages (Table S2, Table S3, Table S4); number of proteins related to metabolic pathways (Table S5); number of proteins extracted by SPM-LLE and direct cell lysis (Table S7) (PDF)
