# Supplementary material for: Proteome Coverage after Simultaneous Proteo-Metabolome Liquid–Liquid Extraction
Source: J Proteome Res. 2023 Feb 10;22(3):951–66. doi: 10.1021/acs.jproteome.2c00758 (PMC9990123; doi:10.1021/acs.jproteome.2c00758)
Supplement: Supplementary file 1 — pr2c00758_si_001.pdf [file pr2c00758_si_001.pdf]

Proteome coverage after simultaneous proteo-metabolome liquid-liquid extraction

Alienke van Pijkeren‡ [1,2], Anna-Sophia Egger‡ [1], Madlen Hotze [1], Elisabeth Zimmermann [1], Tobias Kipura [1], Julia Grander [3], André Gollowitzer [3], Andreas Koeberle [3], Rainer Bischoff* [2], Kathrin Thedieck* [1,4,5], Marcel Kwiatkowski* [1]

[1] Institute of Biochemistry and Center for Molecular Biosciences Innsbruck, University of Innsbruck, Innsbruck, Austria

[2] Department of Analytical Biochemistry and Interfaculty Mass Spectrometry Center, Groningen Research Institute of Pharmacy, University of Groningen, Groningen, the Netherlands

[3] Michael Popp Institute and Center for Molecular Biosciences Innsbruck (CMBI), University of Innsbruck, 6020, Innsbruck, Austria

[4] Laboratory of Pediatrics, Section Systems Medicine of Metabolism and Signaling, University of Groningen, University Medical Center Groningen, Groningen, the Netherlands.

[5] Department for Neuroscience, School of Medicine and Health Sciences, Carl von Ossietzky University Oldenburg, Oldenburg, Germany

‡ The authors contributed equally
* Corresponding authors

E-mail: marcel.kwiatkowski@uibk.ac.at

Table of content

Figure S1: Localization and physicochemical properties of proteins identified in SPM-LLE: P. 2
Figure S2: Relative abundances of all proteins quantified in SPM-LLE: P. 3
Figure S3: GO enrichment of proteins extracted more efficiently with SDC, SDS or urea: P. 4
Figure S4: Relative abundancies related to specific KEGG pathways: P. 5
Figure S5: Comparison of identified proteins that are related to metabolic pathways: P. 6
Figure S6: Extraction efficiencies for proteins related to metabolic pathways: P. 7
Figure S7: Quantitative comparison of proteins extracted by direct cell lysis using urea or SDC: P. 8
Figure S8: GO analysis of proteins extracted more efficiently by direct cell lysis or SPM-LLE: P. 9
Figure S9: GO analysis of proteins extracted more efficiently by urea (SPM-LLE vs. direct cell lysis): P. 10
Figure S10: Differential analysis of metabolic proteins using SDC (SPM-LLE vs. direct cell lysis): P. 11
Figure S11: Differential analysis of metabolic proteins using SDS (SPM-LLE vs. direct cell lysis): P. 12
Figure S12: Differential analysis of metabolic proteins using urea (SPM-LLE vs. direct cell lysis): P. 13
Table S1: Number of reproducibly identified proteins using SPM-LLE: P. 14
Table S2: Relative percentage of missed cleavages using SDC: P. 14
Table S3: Relative percentage of missed cleavages using SDS: P. 14
Table S4: Relative percentage of missed cleavages using urea: P. 15
Table S5: Number of proteins related to metabolic pathways extracted by SPM-LLE: P. 15
Table S6: Number of reproducibly identified proteins using SPM-LLE: P. 15
Table S7: Number of proteins extracted from SPM-LLE vs direct cell lysis: P. 16

Supplemental Figures

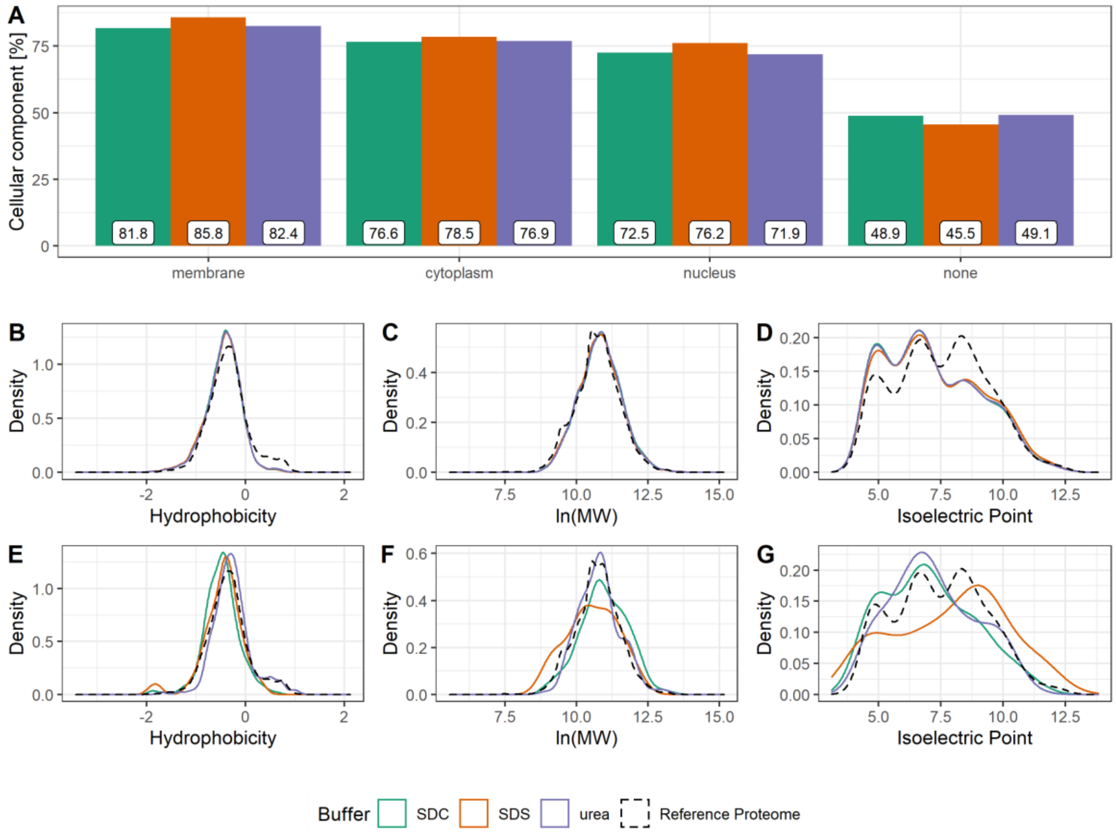

Figure S1: Localization and physicochemical properties of proteins identified in simultaneous proteo-metabolomic liquid-liquid extraction (SPM-LLE) interphase pellets. A: Distribution of proteins across the main GO cellular component categories (membrane proteins, GO: 0016020; nuclear proteins, GO: 0005634; cytoplasmatic proteins, GO: 0005737). B, C, D: Physicochemical properties (hydrophobicity (B), molecular weight (C), isoelectric point (D)) of all proteins identified with the three extractants (sodium deoxycholate (SDC, green), sodium dodecyl sulfate (SDS, orange), urea (purple)). E, F, G: Physicochemical properties (hydrophobicity (E), molecular weight (F), isoelectric point (G)) of proteins identified exclusively upon extraction with SDS (orange), SDC (green) or urea (blue) extracts. Dashed line, human reference proteome (SwissProt, uniprot.org).

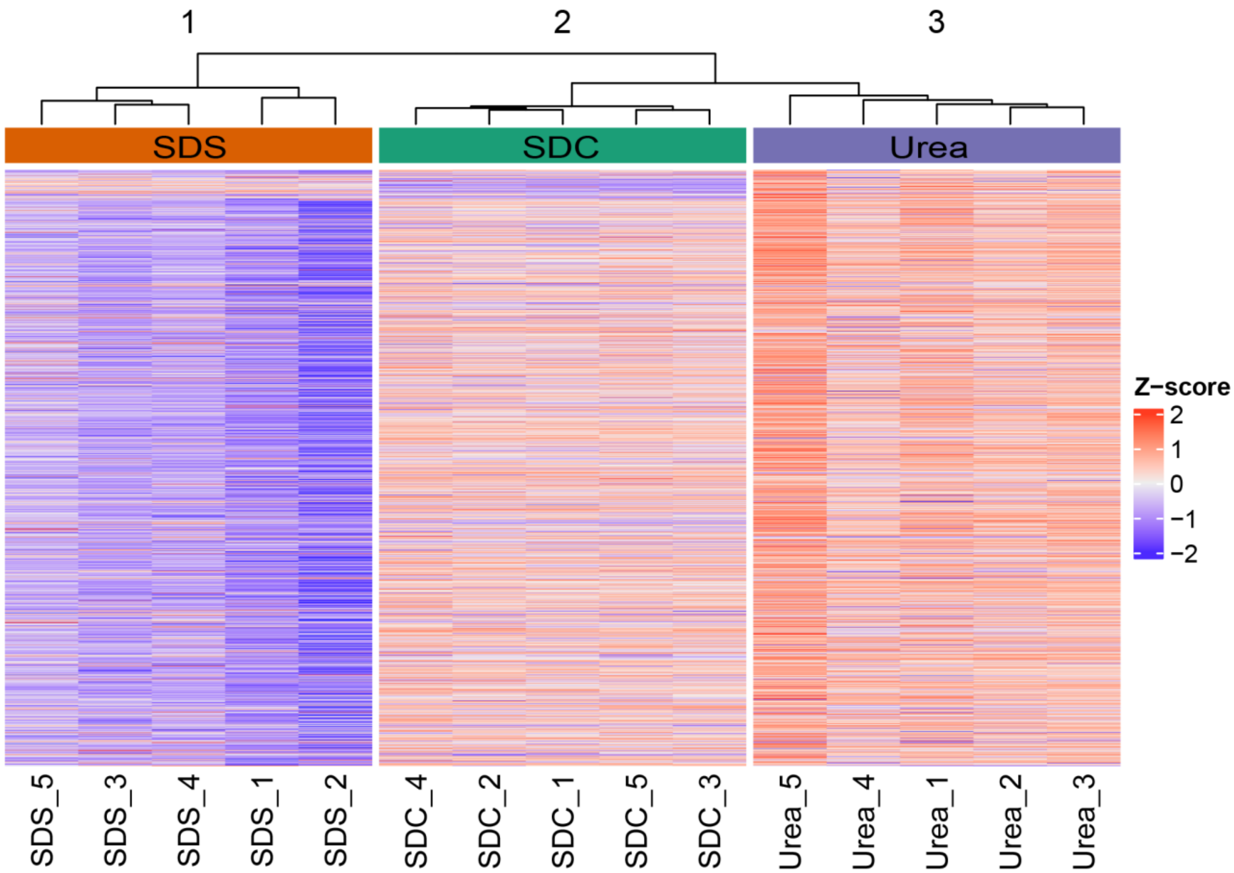

Figure S2: Relative abundances of all proteins quantified in simultaneous proteo-metabolomics liquid-liquid extraction interphase pellets extracted by sodium deoxycholate (SDC, green), sodium dodecyl sulfate (SDS, orange), urea (purple) based buffer systems. n= 5 independent experiments (biological replicates).

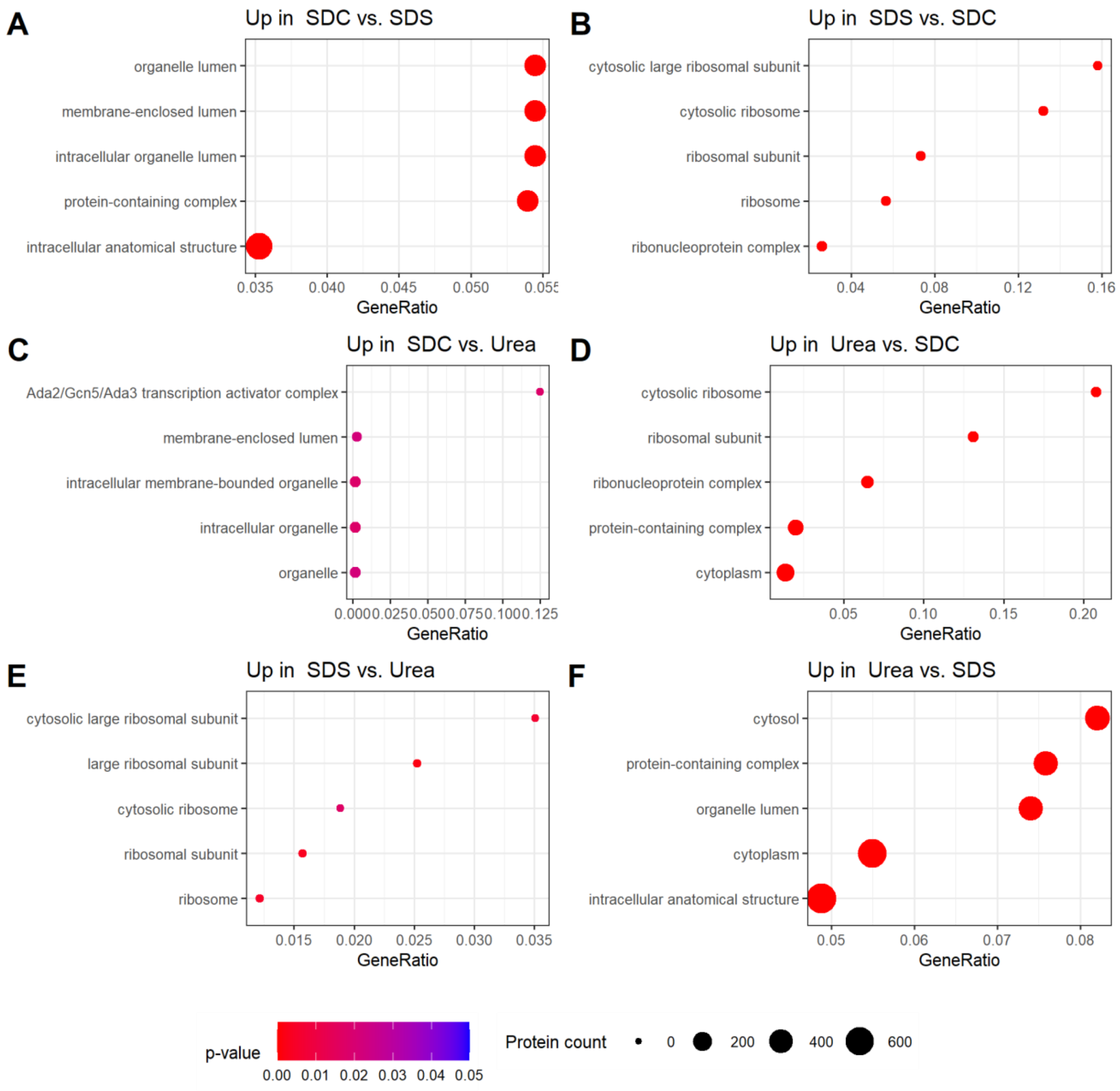

Figure S3: Gene-Ontology (GO)-enrichment (cellular component) of proteins extracted more efficiently with sodium deoxycholate (SDC), sodium dodecyl sulfate (SDS) or urea. Proteins are considered to be extracted more efficiently if they showed a fold-change of ≥ 1.5 and an adjusted *p*-value ≤ 0.05 (see Figure 3B). The size of the dots indicates the protein count of enriched proteins related to the specific GO-term. n= 5 independent experiments (biological replicates).

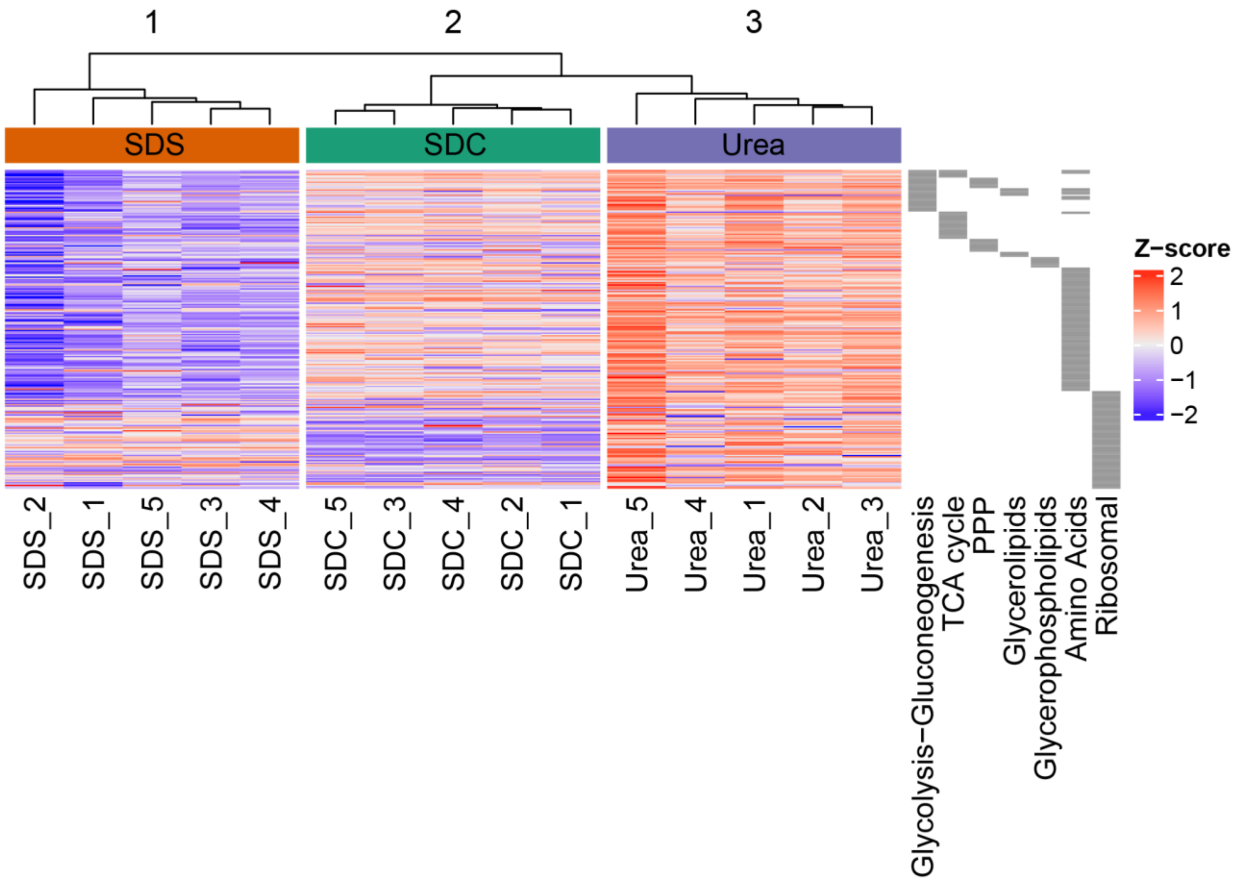

Figure S4: Relative abundancies related to specific KEGG pathways for proteins identified from interphase pellets extracted with SDS (green), SDC (orange) or urea (purple). Glycolysis-Gluconeogenesis: hsa00010; TCA cycle: hsa00020; Phosphate Pentose Pathway (PPP): hsa00030; Glycerolipids: hsa00561; Glycerophospholipids: hsa00564; Amino Acids: hsa00220, hsa00250, hsa00260, hsa00270, hsa00280, hsa00290, hsa00300, hsa00310, hsa00330, hsa00340, hsa00350, hsa00360, hsa00380, hsa00400; Ribosomal Proteins: hsa03010. n= 5 independent experiments (biological replicates).

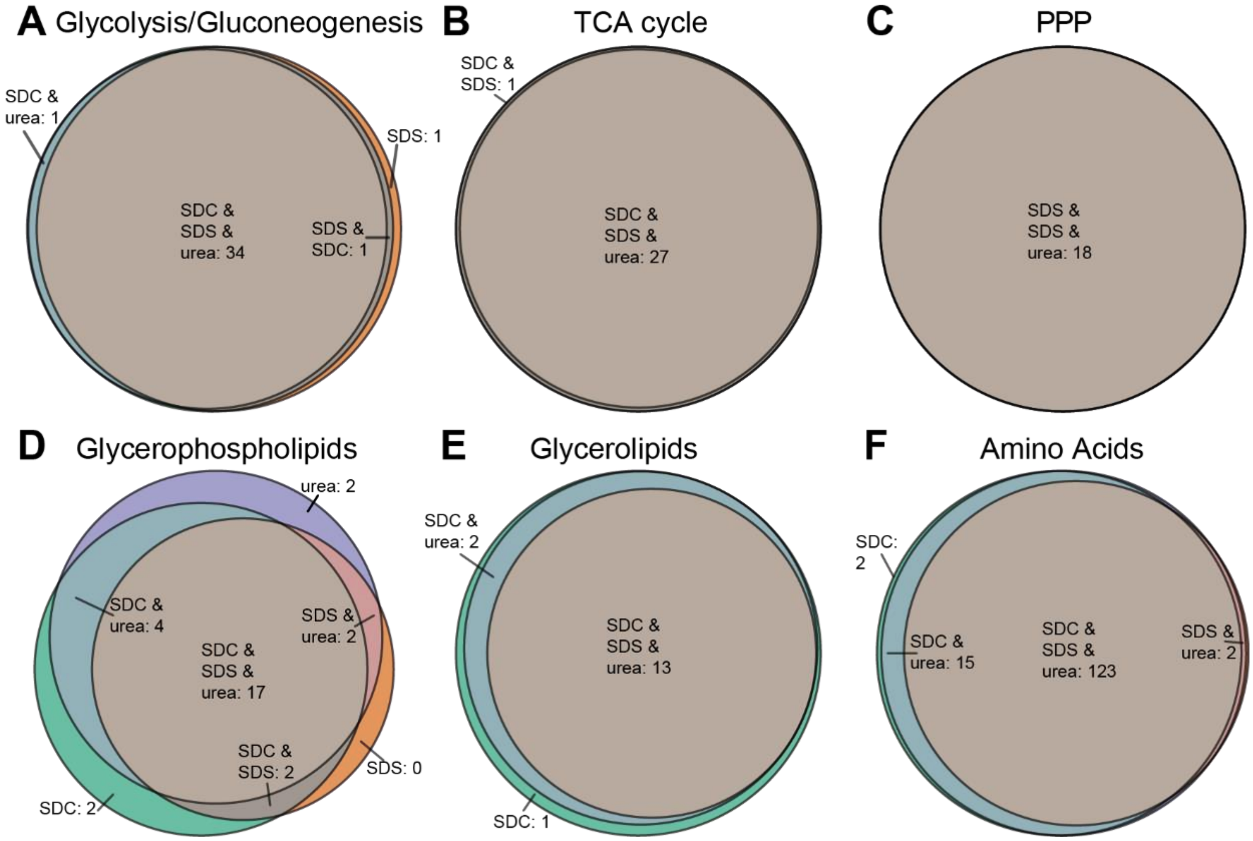

Figure S5: Comparison of identified proteins that are related to metabolic pathways. Number of proteins reproducibly identified in all independent experiments (n = 5, biological replicates).

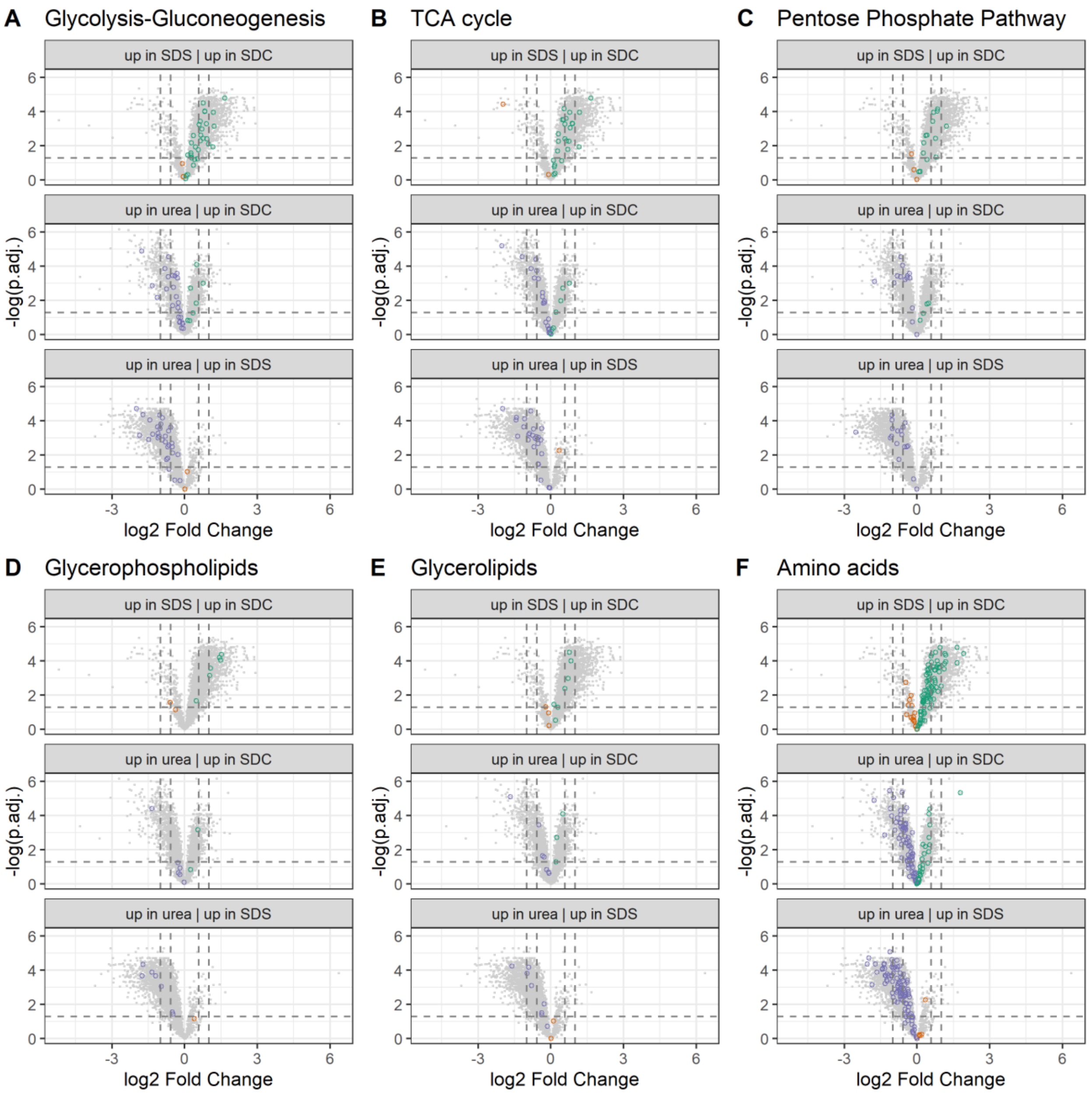

Figure S6: Extraction efficiencies for proteins related to metabolic pathways from interphase pellets extracted with sodium deoxycholate (SDC), sodium dodecyl sulfate (SDS) or urea. Proteins that are associated with glycolysis/gluconeogenesis, the tricarboxylic acid cycle (TCA cycle), pentose phosphate pathway, glycerophospholipids, glycerolipids and amino acids metabolism based on KEGG pathways are highlighted dependent on the extraction agent in purple (urea), green (SDC) and orange (SDS). Vertical line: threshold adjusted *p*-value: 0.05. Two horizontal lines: fold change (FC) threshold: 1.5 and >2. n= 5 independent experiments (biological replicates).

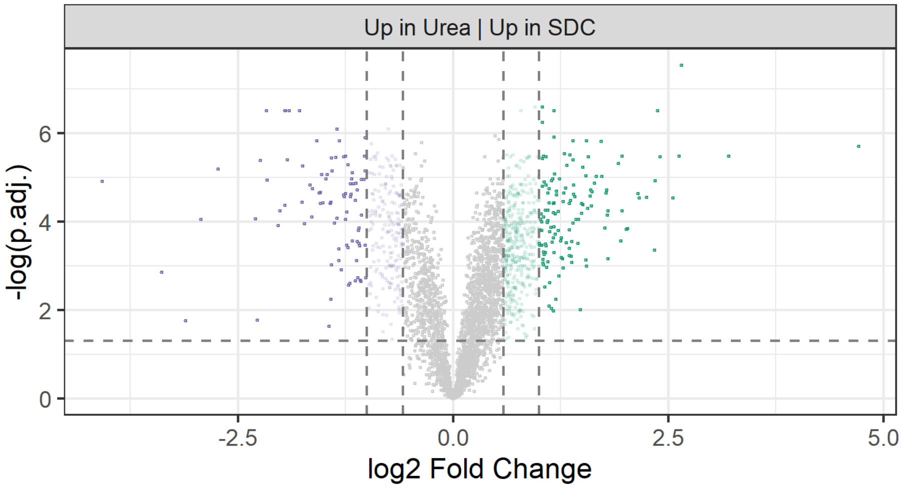

Figure S7: Quantitative comparison of proteins extracted by direct cell lysis using urea (purple) or SDC (green). Significance threshold for enrichment: adjusted *p*-value ≤ 0.05 (two-tailed unpaired *t*-test, Benjamini-Hochberg correction), fold change (FC) of 1.5: colored transparent dots, FC of ≥ 2: colored dots).

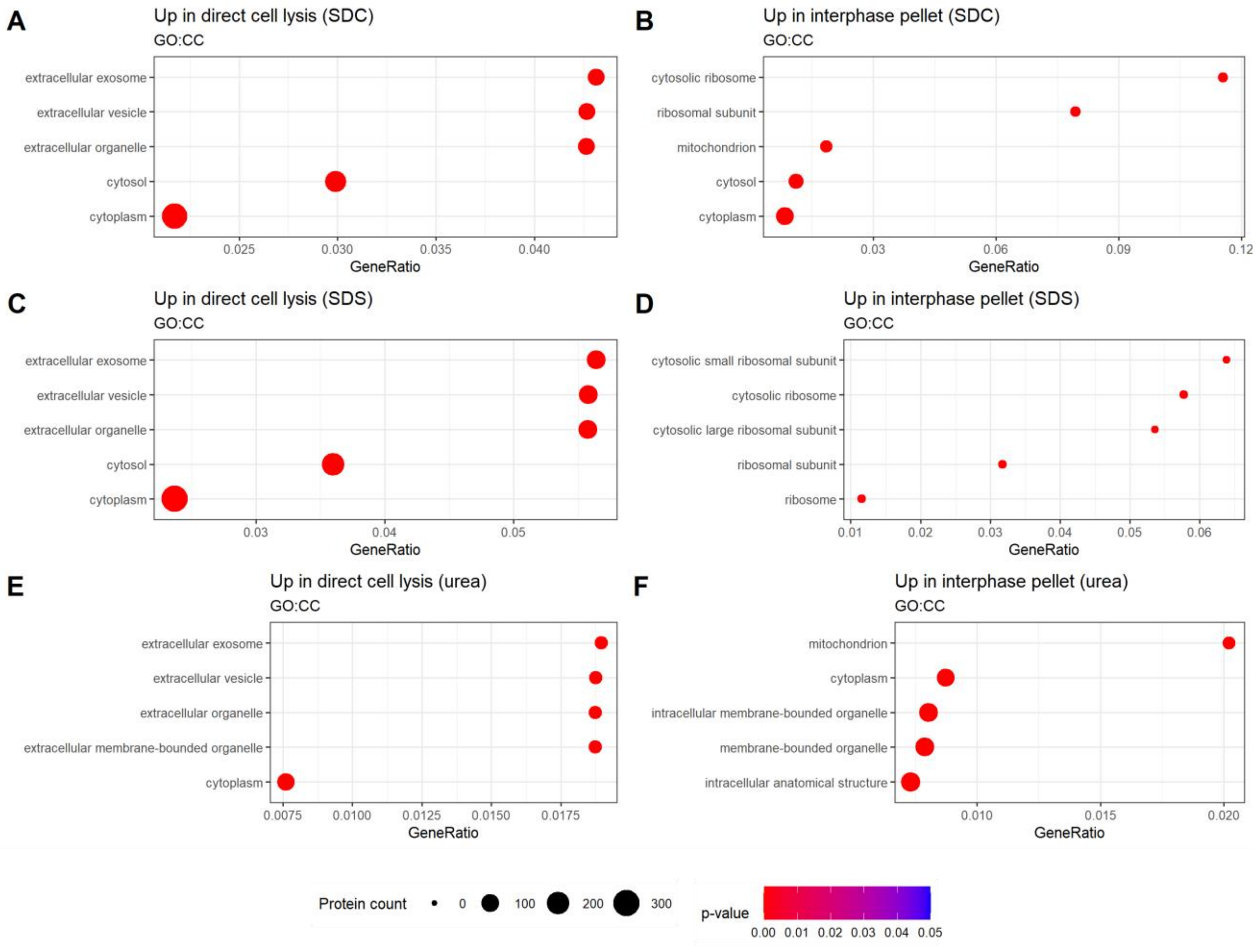

Figure S8: Gene Ontology (GO) enrichment analysis of cellular component (GO:CC) of proteins extracted significantly more efficiently by direct cell lysis (up in cells) versus SPM-LLE interphase pellet. Proteins were considered extracted significantly more efficiently enriched with a FC $\geq$ 2 and a *p*-value $\leq$ 0.05. The size of the dots indicates the number of enriched proteins related to the specific GO-term. n= 5 independent experiments (biological replicates).

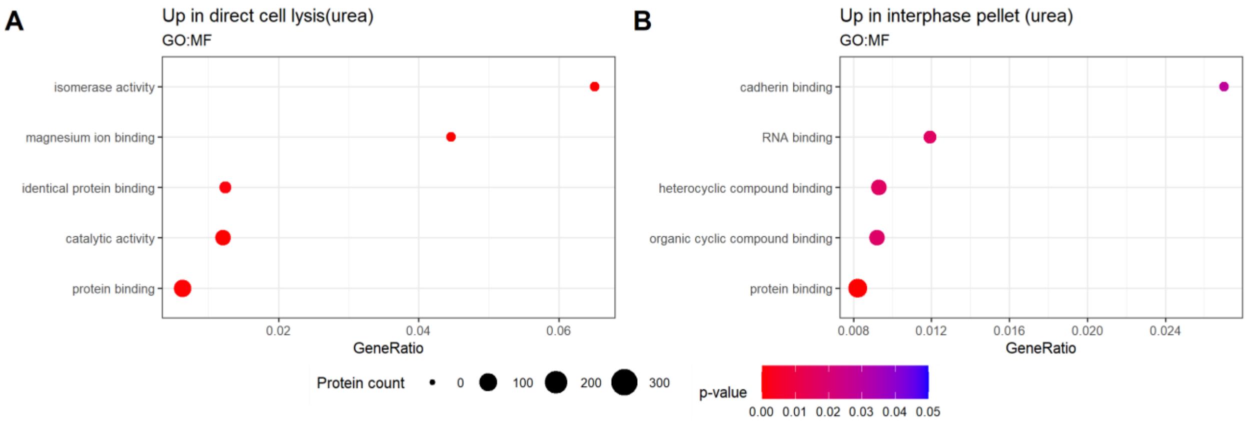

Figure S9: Gene Ontology (GO) enrichment analysis of molecular function (GO:MF) of proteins extracted significantly more efficiently by direct cell lysis (up in cells) versus SPM-LLE interphase pellet using urea. Proteins were considered extracted significantly more efficiently enriched with a FC ≥ 2 and a *p*-value ≤ 0.05. The size of the dots indicates the number of enriched proteins related to the specific GO-term. n= 5 independent experiments (biological replicates).

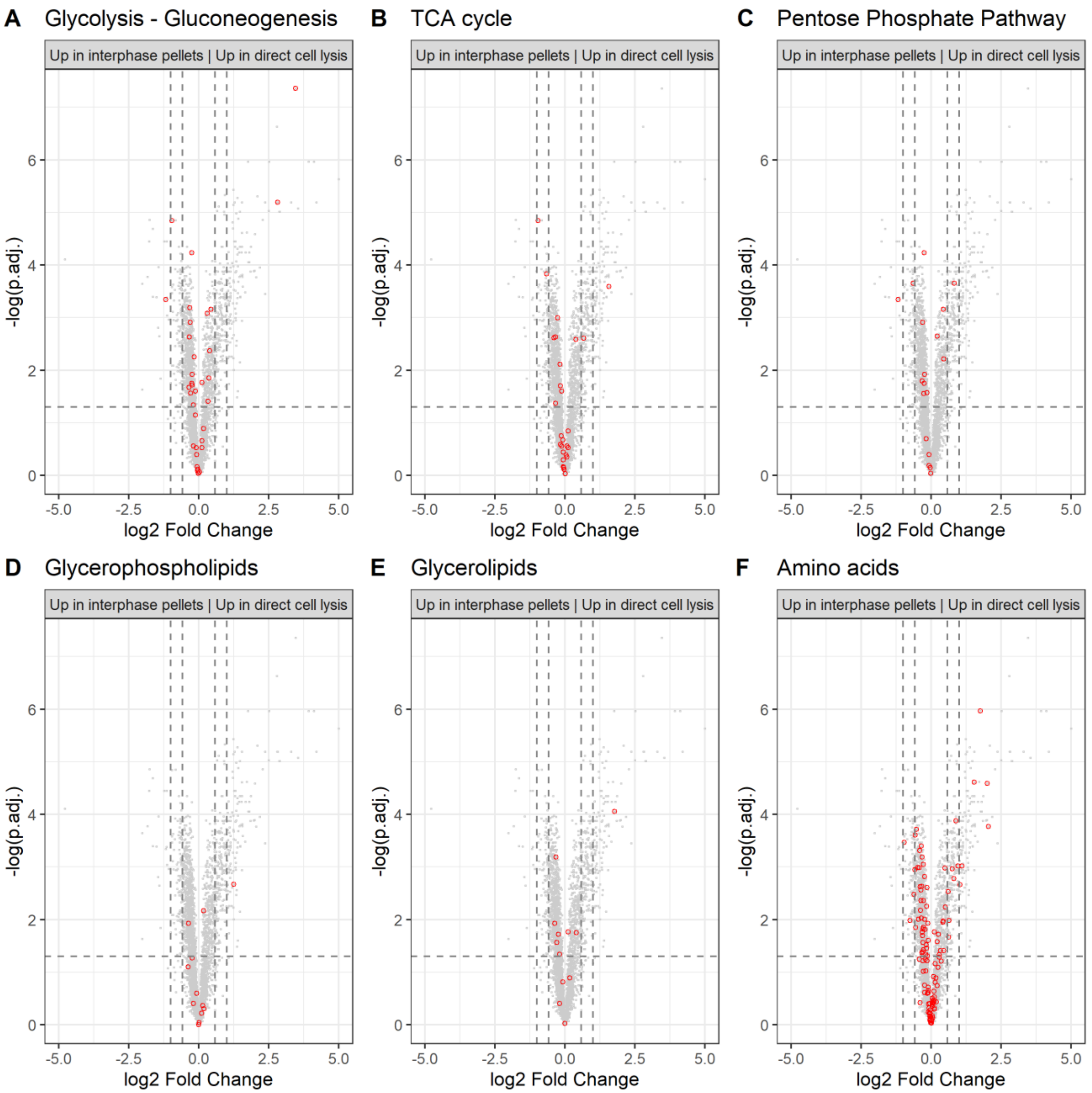

Figure S10: Differential analysis of extraction efficiencies for metabolic proteins using SDC to solubilize SPM-LLE interphase pellets (interphases) versus direct cell lysis (cells). Proteins that are associated with glycolysis/gluconeogenesis, the tricarboxylic acid cycle (TCA cycle), pentose phosphate pathway, glycerophospholipids, glycerolipids and amino acids metabolism based on KEGG pathways are highlighted in red circles. Vertical line: threshold adjusted *p*-value: 0.05. Two horizontal lines: fold change (FC) threshold: 1.5 and >2. n= 5 independent experiments (biological replicates).

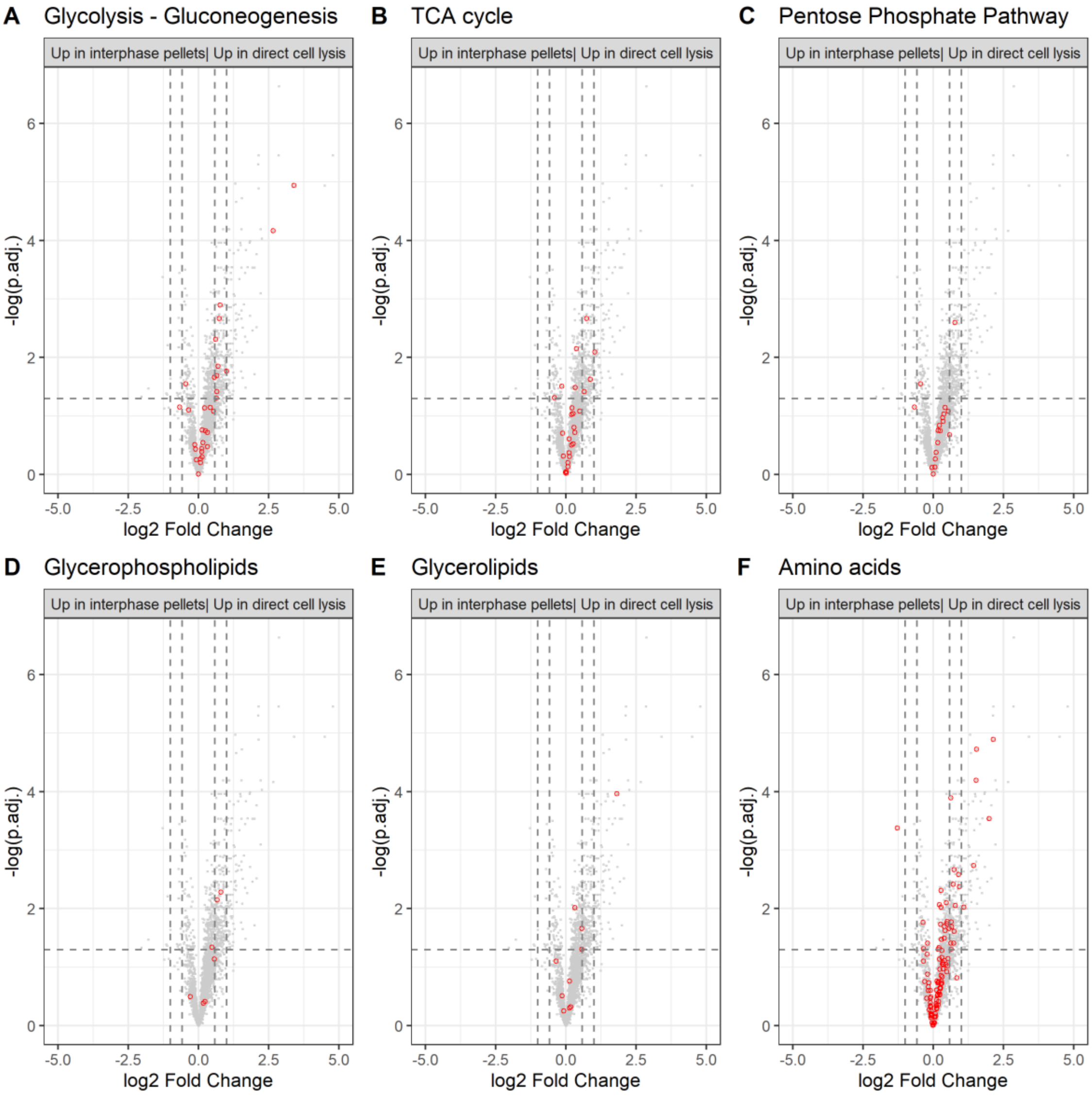

Figure S11: Differential analysis of extraction efficiencies for metabolic proteins using SDS to solubilize SPM-LLE interphase pellets (interphases) versus direct cell lysis (cells). Proteins that are associated with glycolysis/gluconeogenesis, the tricarboxylic acid cycle (TCA cycle), pentose phosphate pathway, glycerophospholipids, glycerolipids and amino acids metabolism based on KEGG pathways are highlighted in red circles. Vertical line: threshold adjusted *p*-value: 0.05. Two horizontal lines: fold change (FC) threshold: 1.5 and >2. n= 5 independent experiments (biological replicates).

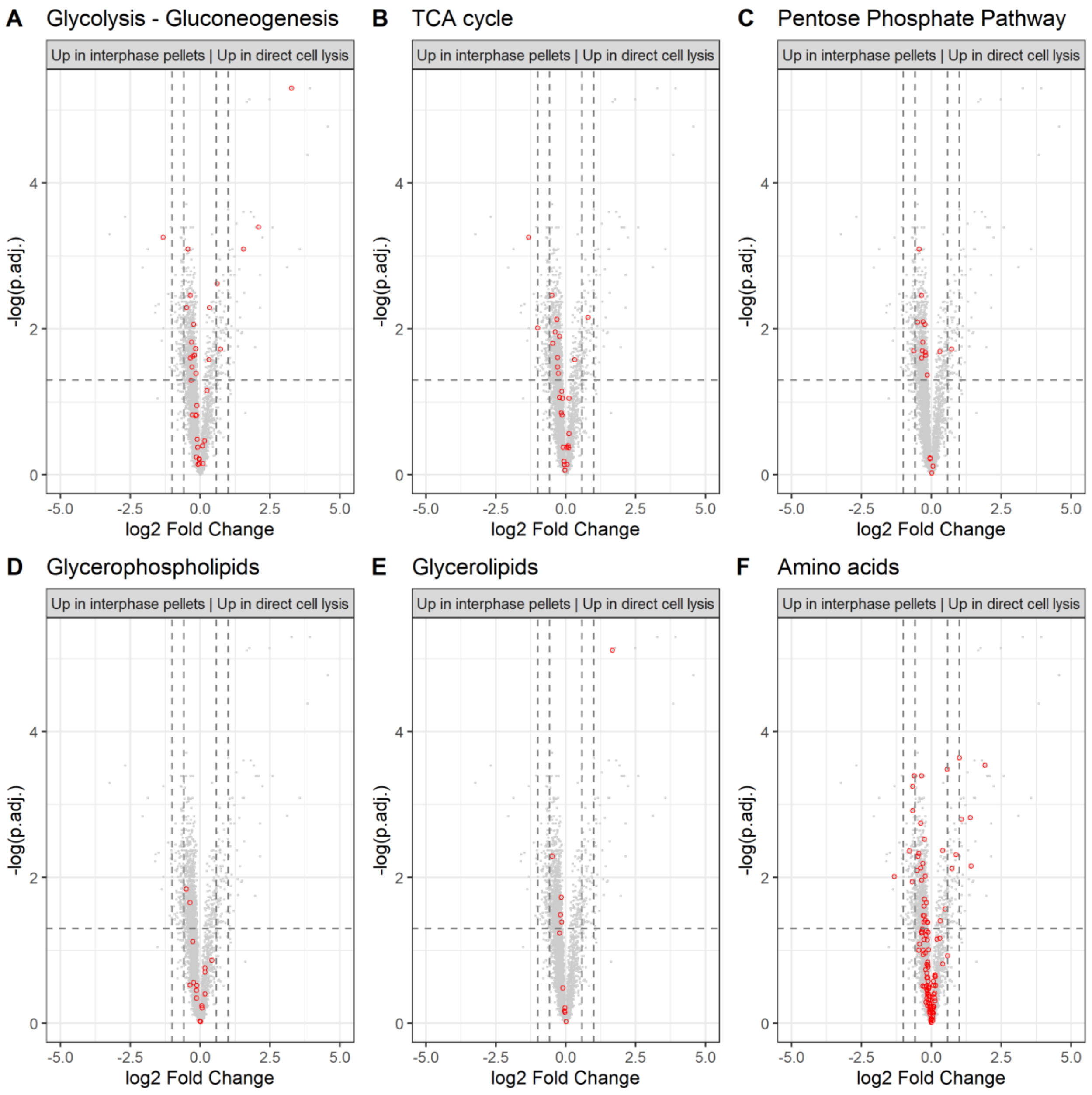

Figure S12: Differential analysis of extraction efficiencies for proteins related to metabolic pathways using urea to solubilize SPM-LLE interphase pellets (interphases) versus direct cell lysis (cells). Proteins that are associated with glycolysis/gluconeogenesis, the tricarboxylic acid cycle (TCA cycle), pentose phosphate pathway, glycerophospholipids, glycerolipids and amino acids metabolism based on KEGG pathways are highlighted in red circles. Vertical line: threshold adjusted *p*-value: 0.05. Two horizontal lines: fold change (FC) threshold: 1.5 and >2. n= 5 independent experiments (biological replicates).

Supplemental Tables

Table S1: Number of reproducibly identified proteins in the proteomes extracted by urea, sodium deoxycholate (SDC) and sodium dodecyl sulfate (SDS) from the SPM-LLE interphase protein pellet. Urea+SDC+SDS: number of identified proteins identified in both urea, SDC and SDS. Urea+SDC: number of identified proteins identified in both urea and SDC. Urea+SDS: number of identified proteins identified in both urea and SDS. SDC+SDS: number of identified proteins identified in both SDC and SDS. Urea: number of proteins identified in urea only. SDC: number of proteins identified in SDC only. SDS: number of proteins identified in SDS only.

Condition	Number of identified protein groups	Percentage
Urea+SDC+SDS	4200	75.5
Urea+SDC	795	14.3
Urea+SDS	38	0.7
SDC+SDS	63	1.1
Urea	199	3.6
SDC	242	4.3
SDS	29	0.5

Table S2: Relative percentage of missed cleavages during tryptic digestion after proteome extraction from SPM-LLE interphase pellet (SPM-LLE interphase pellet) and after proteome extraction by direct cell lysis (Direct cell lysis) using sodium deoxycholate (SDC).

Number of missed cleavages	SPM-LLE interphase pellet [%]	Direct cell lysis [%]
0	64.2 ±0.6	75.9 ±0.9
1	28.9 ±0.4	20.9 ±0.6
2	6.8 ±0.2	3.1 ±0.3

Table S3: Relative percentage of missed cleavages during tryptic digestion after proteome extraction from SPM-LLE interphase pellet (SPM-LLE interphase pellet) and after proteome extraction by direct cell lysis (Direct cell lysis) using sodium dodecyl sulfate (SDS).

Number of missed cleavages	SPM-LLE interphase pellet [%]	Direct cell lysis [%]
0	50.8 ±3	60.3 ±2.7
1	36.2 ±1.5	30.8 ±1.7
2	13 ±1.6	8.9 ±1.1

Table S4: Relative percentage of missed cleavages during tryptic digestion after proteome extraction from SPM-LLE interphase pellet (SPM-LLE interphase pellet) and after proteome extraction by direct cell lysis (Direct cell lysis) using urea.

Number of missed cleavages	SPM-LLE interphase pellet [%]	Direct cell lysis [%]
0	75.1 ±1.3	69.6 ±3.1
1	21.6 ±0.9	25.4 ±2.1
2	3.3 ±0.4	5 ±1

Table S5: Number of proteins related to metabolic pathways extracted from SPM-LLE interphase pellet with at least 1.5-fold higher efficiency.

	SDS vs. SDC		urea vs. SDC		urea vs. SDS	
	# proteins up in SDS	# proteins up in SDC	# proteins up in urea	# proteins up in SDC	# proteins up in urea	# proteins up in SDS
Glycolysis/gluconeogenesis	0	16	7	1	22	0
Tricarboxylic acid cycle	1	11	5	1	15	0
Pentose phosphate pathway	0	7	6	0	11	0
Glycerophospholipids metabolism	1	5	1	0	5	0
Glycerolipid metabolism	0	3	1	0	4	0
Amino acid metabolism	0	37	20	1	59	0

Table S6: Number of reproducibly identified proteins in the proteomes extracted by direct cell lysis using urea, sodium deoxycholate (SDC) and sodium dodecyl sulfate (SDS). Urea+SDC+SDS: number of identified proteins identified in both urea, SDC and SDS. Urea+SDC: number of identified proteins identified in both urea and SDC. Urea+SDS: number of identified proteins identified in both urea and SDS. SDC+SDS: number of identified proteins identified in both SDC and SDS. Urea: number of proteins identified in urea only. SDC: number of proteins identified in SDC only. SDS: number of proteins identified in SDS only.

Condition	Number of identified protein groups	[%]
SDS+SDC+Urea	4646	82.8
SDC+Urea	423	7.5
Urea+SDS	58	1.0
SDC+SDS	80	1.4
Urea	113	2.0
SDC	241	4.3
SDS	48	0.9

Table S7: Number of proteins extracted from SPM-LLE interphase pellets and by direct cell lysis with at least 1.5-fold higher efficiency.

	**Urea vs. SDC**	
	# proteins up in urea	# proteins up in SDC
Direct cell lysis	357	632
SPM-LLE interphase pellets	488	167
